# A taxonomic revision of the *silphaeformis* species-group of the genus *Tachinus* Gravenhorst (Staphylinidae, Tachyporinae) from China

**DOI:** 10.3897/zookeys.357.5861

**Published:** 2013-12-05

**Authors:** Ting Feng, Michael Schülke, Li-Zhen Li

**Affiliations:** 1 Department of Biology, College of Life and Environmental Sciences, Shanghai Normal University, Shanghai, P. R. China; 2 Blankenfelder Straße 99, 13127 Berlin, Germany

**Keywords:** *Tachinus*, *silphaeformis* species group, revision, key, China, distributional maps

## Abstract

The Chinese species of the *silphaeformis* group of the genus *Tachinus* Gravenhorst are revised with fifteen species being treated. Thirteen of them are described as new: *T. armatus* Feng & Li, **sp. n.** (Sichuan), *T. cavazzutii* Feng, Li & Schülke, **sp. n.** (Sichuan), *T. coronatus* Feng, Li & Schülke, **sp. n.** (Ningxia, Qinghai), *T. hercules* Feng, Li & Schülke, **sp. n.** (Sichuan), *T. hujiayaoi* Feng, Li & Schülke, **sp. n.** (Shaanxi), *T. jiuzhaigouensis* Feng, Li & Schülke, **sp. n.** (Sichuan), *T. linzhiensis* Feng & Li, **sp. n.** (Tibet), *T. maderianus* Feng & Li, **sp. n.** (Sichuan), *T. mengdaensis* Feng, Li & Schülke, **sp. n.** (Qinghai), *T. oblongoelytratus* Feng & Li, **sp. n.** (Sichuan), *T. parahercules* Feng, Li & Schülke, **sp. n.** (Sichuan), *T. paralinzhiensis* Feng & Li, **sp. n.** (Tibet), and *T. yini* Feng, Li & Schülke, **sp. n.** (Sichuan). The two known species are redescribed based on the holotypes and additional material. Illustrations of the habitus and major diagnostic characters, distributional maps, and identification keys of all species are included.

## Introduction

The genus *Tachinus* Gravenhorst, 1802 currently contains more than 250 valid species, most of them distributed in the Holarctic and Oriental regions, with a few reaching the Neotropics in Central America (Herman 2001). Members of *Tachinus* from China are assigned to three subgenera, viz., *Tachinus* Gravenhorst, 1802, *Tachinoderus* Motschulsky, 1858, and *Latotachinus* Ullrich, 1975.

The *silphaeformis* group is one of the 30 species groups of the subgenus *Tachinus* and includes seven described species (Ullrich 1975; Schülke 1997): *Tachinus silphaeformis* Normand, 1928 from Tunisia, *Tachinus mercatii* Jarrige, 1966 from Italy, *Tachinus starcki* Eppelsheim, 1889 from Russia (West Caucasus), *Tachinus alienus* Ullrich, 1975 from northern India, *Tachinus lacinipennis* (Scheerpeltz, 1976) from Nepal, and *Tachinus lohsei* Ullrich, 1975 and *Tachinus maderi* Bernhauer, 1939 from southwestern China.

In this paper, we aim to revise the Chinese species of the *silphaeformis* group. This includes redescriptions of the two known species and descriptions of thirteen new species. Illustrations of the habitus and major diagnostic features, identification keys, and distributional maps of all species are provided.

## Material and methods

More than 1,050 specimens of the *silphaeformis* group were examined. Material from the following collections was used for study:

FMNH Field Museum of Natural History, Chicago

SNUC Insect Collection of Shanghai Normal University, Shanghai

MHNG Museum d'Histoire Naturelle de Genève

NMP National Museum Praha

NHMB Natural History Museum Basel

cAss Private Collection of Volker Assing, Hannover

cFel Private Collection of Benedikt Feldmann, Münster

cSme Private Collection of Aleš Smetana, Ottawa

cSch Private Collection of Michael Schülke, Berlin

cPüt Private Collection of Andreas Pütz, Eisenhüttenstadt

The dissection procedure was the following: dried specimens were immersed in cold water for 0.5 to 1 hour. After relaxing, the membrane between abdominal segments VI and VII was cut. The dissected parts were mounted in Euparal (Chroma Gesellschaft Schmidt, Koengen, Germany) on a plastic slide.

Habitus photos were taken using a Cannon EOS 40D camera mounted with an MP-E 65 mm Macro Photo Lens. Photos of dissected parts were taken using a Cannon G9 camera mounted on an Olympus CX21 microscope. Drawings and distribution maps were made in Adobe Illustrator CS5.

All measurements are in milimeters. Measurements were made based on the holotype and a random sample of specimens available for each species (holotype in brackets). Relative lengths of the antennomeres are given from single specimens.

The following abbreviations are used in the text:

BL: body length, from the anterior margin of the head to the posterior margin of the abdominal tergite VIII; FL: forebody length, from the anterior margin of the head to the posterior margin of the elytra; PL: length of the pronotum along the midline; EL: length of the elytra along the suture; SEL: longitudinal distance from the posterior sutural angle to level of the elytral apex; HW: width of the head across the eyes; PW: maximum width of the pronotum; EW: maximum width of the elytra.

The holotypes of the new species are deposited in **SNUC**.

## Taxonomy

### *silphaeformis* species-group

*silphaeformis* species-group Ullrich, 1975: 273.

**Description.** Body brown to black, elytra and/or pronotal lateral margins yellowish to pale brownish, surface shiny, elytra sometimes dull; mouthparts and legs reddish brown or yellowish brown, basal four antennomeres sometimes yellowish or pale brownish.

Moderate in size; BL in male: 3.56–4.95; FL in male: 2.61–3.78. Female larger than male, or of similar size, BL: 3.57–5.12; FL: 2.82–4.11.

Body narrowly to broadly elongate-oval, with sides of abdomen evenly narrowing from base to apex. Surfaces of head, pronotum, elytra and abdomen with dense microsculpture and punctation.

Head nearly sub-triangular, shorter than wide. Eyes moderately large. Ocular setae distinct. Maxillary palpi long, robust, palpomere I short, II longer, III shorter than II, palpomere IV conical, more than twice as long as III. Labial palpi short, three-segmented, with palpomere I long, II much shorter, III as long as I. Antennae with basal four antennomeres lacking fine recumbent pubescence, antennomeres V–XI usually with recumbent pubescence; XI longer than X, with apex narrowly rounded.

Pronotum transverse, PL: PW = 0.53–0.75 in male, 0.57–0.76 in female.

Elytra long, EL: PL = 1.49–1.73 in male, 1.50–1.93 in female. Wings fully developed.

Abdominal tergites III–VI each with one pair of pruinose spots near middle, posterior margin of tergite VII with distinct palisade fringe.

Body length, elytral length, elytral microsculpture and shape of posterior margin usually subject to sexual dimorphism.

Male. Elytra shorter, posterior margin without modifications, sutural angle simple; elytral microsculpture often weak. Protarsomeres I–IV strongly dilated; sternite VII (Figs 9–10) with apical margin emarginate, in most species bent ventrad at middle, with large area covered with coarse granules; tergite VIII (Figs 11–12) with sinuate margin or three to four rounded lobes; posterior margin of male sternite VIII (Figs 13–14) with deep bell-shaped emargination; aedeagus (Figs 15–17) with median lobe moderately short and broad, parameres fused at base, of variable shape, often of reduced length, and of latero-apicad orientation.

Female. Elytra longer, posterior margin simply rounded, truncate, slightly to strongly produced at sutural angle; SEL distinctly longer than in male; elytral microsculpture more distinct. Protarsomeres I–IV not dilated; tergite VIII (Figs 18–19) with sinuate apical margin, or with two to three rounded lobes; female sternite VIII (Fig. 20) with six lobes, fimbriate median lobes sometimes fused.

**Remarks.** Members of this group can be readily separated from those of the other groups by the long and sexually dimorphic elytra, the presence of a pair of pruinose spots on tergites III–VI, the characterically impressed and apically bent male sternite VII, and the unique shape of aedeagus. Most species of this group are very similar in general appearance, except for *Tachinus lohsei* and *Tachinus jiuzhaigouensis*, which are distinguished by their small size and yellow lateral margins of the pronotum. Reliable identifications of the group members require careful examinations of the genital features including the form of the tergite and sternite VIII of both sexes, and the aedeagus.

Data on the natural history is largely unknown for most species. Many species of this group are restricted to higher elevations, mostly above 2,500 m. They were collected, often together with other *Tachinus* species from sifted litter, dung, and decaying mushrooms in mixed coniferous forests, above the forest margin from litter of *Rhododendron* and other subalpine shrubs, or from dung on alpine meadows.

### Tachinus (s. str.) armatus

Feng, Li & Schülke sp. n.

http://zoobank.org/0883CBB7-AAE8-4D29-BB48-1B687F407B79

http://species-id.net/wiki/Tachinus_armatus

Figs 1A , 1B , 9A , 10A , 11A , 13A , 15A , 18A , 20A , 21A

#### Type locality.

China, Sichuan, Nanping, Jiuzhaigou Natural Reserve.

#### Type material.

**Holotype**: ♂, **CHINA**: Sichuan Prov., Aba A. R., Nanping County, Jiuzhaigou, 27.vii.2001, LI & ZHAO leg. (SNUC). **Paratypes**: 1 ♀, same label data as holotype (SNUC); 1 ♂, 1 ♀, **CHINA**: N-Sichuan Pass btw. Songpan & Nanping, E side, 3450–3500 m, 21.VI.2002, leg. S. Murzin & I. Shokhin (cSch).

#### Description.

Measurements of holotype: BL 4.00;FL 3.06; PL 0.83; EL 1.45; SEL 0.11; HW 0.83; PW 1.33; EW 1.50; relative length of antennomeres I–XI: 23: 14: 15: 10: 15: 15: 15: 12: 12: 12: 26. Measurements of female paratype: BL 4.89; FL 3.61; FL 3.61; PL 0.89; EL 1.67; SEL 0.28; HW 0.89; PW 1.45; EW 1.72; relative length of antennomeres I–XI: 23: 16: 16: 10: 13: 12: 12: 12: 10: 12: 26.

**Figure 1.**
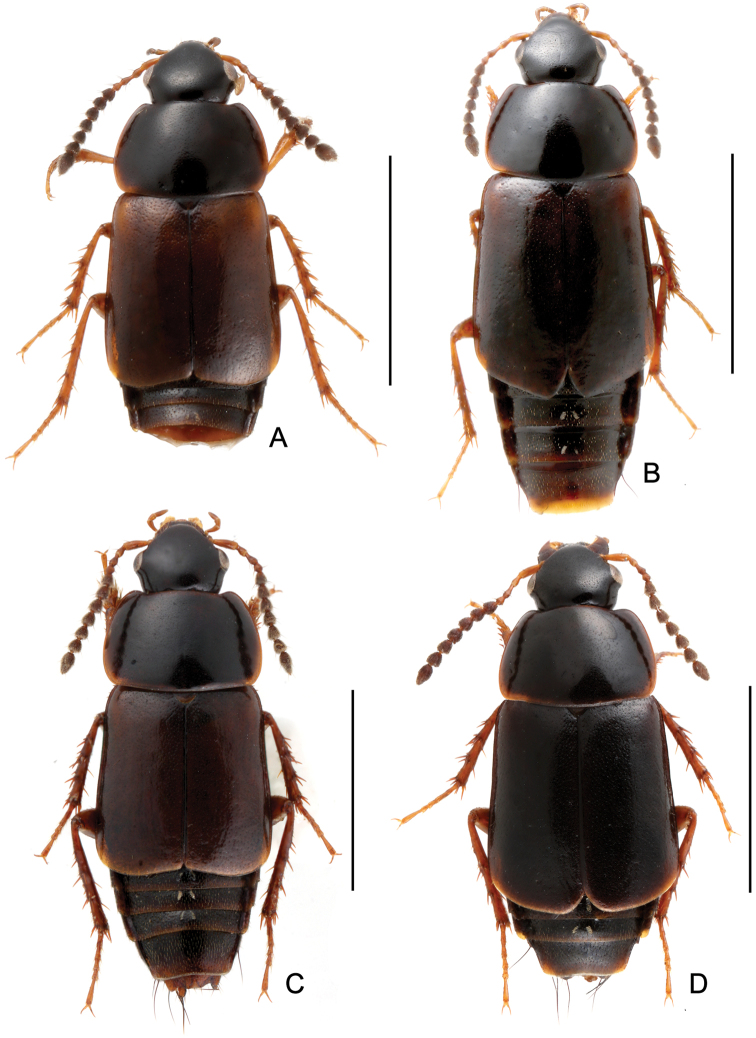
Habitus. **A** *Tachinus armatus*, male holotype **B** *Tachinus armatus*, female **C** *Tachinus cavazzutii*, male holotype **D** *Tachinus cavazzutii*, female. Scales: 2 mm.

Body (Figs 1A, 1B) dark brown; mouthparts and elytra reddish brown; basal four antennomeres and legs reddish yellow.

Head shorter than wide, HW: PW = 0.58–0.62, disc with microsculpture consisting of irregular striae, punctation fine and sparse. Antennae moderately short, antennomeres X slightly shorter than wide.

Pronotum distinctly wider than long (PL: PW = 0.61–0.62); apical margin transversely concave, base slightly convex; surface with microsculpture consisting of irregular transverse striae, punctation slightly finer than that of head.

Elytra slightly elongate; EL: EW = 0.96; EL: PL = 1.73; EW: PW = 1.13 in male; EL: EW = 0.97; EL: PL = 1.88; EW: PW = 1.19 in female. Surface with microsculpture composed of fine meshes, punctation coarser than that of head.

Surface of abdomen with microsculpture radiating from punctures.

Male. Posterior margin of sternite VII (Figs 9A, 10A) broadly and deeply emarginate, apical margin bent ventrad, with moderately broad area of coarse granules. Tergite VIII (Fig. 11A) with four short lobes, median lobes slightly longer than lateral ones. Sternite VIII (Fig. 13A) broad. Median lobe of aedeagus (Fig. 15A) broad and projecting beyond apex of parameres, parameres fused, forming one apically truncate plate.

Female. Body (Fig. 1B) slightly larger than that of male. Elytra only slightly longer than in male; inner part of posterior margin produced, forming a distinct angle. Microsculpture more distinct than in male. Lobes of tergite VIII (Fig. 18A) completely reduced, posterior margin sinuate, lateral angles each with pair of long setae. Sternite VIII (Fig. 20A) with fimbriate median lobes fused and as long as sublateral ones.

#### Etymology.

The specific name (Latin adjective), meaning “armed”, refers to the conspicuous secondary sexual features of the male.

#### Remarks.

Males can be separated from those of the other species by the unique shape of the parameres and of the apex of the median lobe of the aedeagus. Females are distinguished by the shape of tergite VIII, and by the reduced median lobes of the female sternite VIII.

### Tachinus (s. str.) cavazzutii

Feng, Li & Schülke sp. n.

http://zoobank.org/5974F575-1467-4FF9-A334-3FCE17C4A547

http://species-id.net/wiki/Tachinus_cavazzutii

Figs 1C , 1D , 9B , 10B , 11B , 13B , 15B , 18B , 20B , 21B

#### Type locality.

China, Sichuan Province, Nanping, Jiuzhaigou.

#### Type material.

**Holotype**: ♂, **CHINA**: Sichuan Prov., Aba A. R., Nanping County, Jiuzhaigou, 27.vii.2001, LI & ZHAO leg. (SNUC). **Paratypes**: 11 ♂♂, 11 ♀♀, same label data as holotype (SNUC); 3 ♂♂, 1 ♀, **CHINA**: Sichuan Prov., pass between Pingwu and Nanping, 3100 m, 22.viii.1999, Cavazzuti leg. (cSme, cSch); 2 ♀♀, **CHINA**: N–Sichuan [CH12–20] 60 km N Songpan, road S 301 km 103, N Gongangling pass, 33°10'06"N, 103°43'13"E, 3000 m, forest near creek, litter sifted, 9.viii.2012, leg. M. Schülke (cSch); ♂, **CHINA**: N–Sichuan [CH12–19] 47 km N Songpan, road S 301 km 118, N Gongangling pass, 33°03'15"N, 103°43'36"E, 3390 m, spruce forest with shrubs, litter, moss, and mushrooms sifted, 9.viii.2012, leg. M. Schülke (cSch).

#### Description.

Measurements of males (holotype):BL 4.05–4.73 (4.73); FL 3.11–3.78 (3.78); PL 0.89–1.06 (1.06); EL 1.39–1.67 (1.67); SEL 0.10–0.12 (0.12); HW 0.89–1.00 (1.00); PW 1.50–1.67 (1.67); EW 1.78–1.83 (1.83); relative length of antennomeres I–XI: 23: 13: 17: 10: 14: 14: 14: 15: 14: 14: 25. Measurements of females: BL 4.45–4.73; FL 3.45–3.84; PL 1.00–1.06; EL 1.66–1.68; SEL 0.16–0.18; HW 0.89–0.95; PW 1.50–1.61; EW 1.89–1.95. HW: PW = 0.55–0.63; relative length of antennomeres I–XI: 25: 17: 16: 10: 15: 15: 15: 15: 15: 15: 22.

Body (Figs 1C, 1D) dark brown; basal four antennomeres, mouthparts, posterior margin of elytra and legs reddish brown.

Head shorter than wide, HW: PW = 0.53–0.67 (0.60), punctation fine and sparse; microsculpture composed of irregular transverse striae. Antennae moderately short, antennomeres X slightly shorter than wide.

Pronotum: PL: PW = 0.53–0.71 (0.63); apical margin transversely sinuate, base slightly arcuate; side broadly rounded, widest in basal third; microsculpture consisting of irregular transverse striae, punctation denser and finer than that of head.

Elytra only slightly elongate, EL: EW = 0.76–0.91 (0.91), EL: PL = 1.31–1.88 (1.58), EW: PW = 1.07–1.22 (1.10) in males; EL: EW = 0.85–0.89, EL: PL = 1.57–1.68, EW: PW = 1.17–1.30 in females; surface with denser and coarser punctation than that of head and with dense microsculpture consisting of transverse meshes.

Abdomen with microsculpture radiating from punctures.

Male. Sternite VII (Figs 9B, 10B) with posterior margin broadly and deeply emarginate, apical margin bent ventrad, with moderately broad area of coarse granules. Tergite VIII (Fig. 11B) with four almost reduced lobes, lateral lobes slightly longer than median ones. Sternite VIII as in Fig. 13B. Aedeagus (Fig. 15B) with median lobe broad and projecting beyond apices of parameres, apical portion of median lobe with slight projection; parameres short, of latero-apicad orientation, broadly triangular in lateral view.

Female (Fig. 1D). Pronotum with microsculpture more distinct, forming transverse meshes. Elytra distinctly longer than that of male; posterior margin rounded; microsculpture denser and more distinct than in male. Abdominal tergite VIII (Fig. 18B) with posterior margin deeply emarginate medially, lateral angles each with one long seta. Sternite VIII (Fig. 20B) with six distinct lobes, fimbriate median lobes separated by shallow emargination.

#### Etymology.

The species is named after Pierfranco Cavazzuti (Pagno, Italy), the collector of some paratypes.

**Remarks.** Males can be separated from the other species by the apical projection of the median lobe of the aedeagus, and by the broadly triangular parameres (in lateral view). Females are distinguished by the unique shape of tergite VIII.

### Tachinus (s. str.) coronatus

Feng, Li & Schülke sp. n.

http://zoobank.org/6E800F79-DAE0-4D7B-9C4D-247E3EDFCA7C

http://species-id.net/wiki/Tachinus_coronatus

Figs 2A , 2B , 9C , 10C , 11C , 13C , 15C , 18C , 20C , 21C

#### Type locality.

China, Ningxia A. R., Jingyuan, Yehegu Valley.

#### Type material.

**Holotype**: ♂, **CHINA**: Ningxia A. R., Guyuan City, Jingyuan County, Xixia Forestry Station, Yehegu, alt. 1,900 m, 13.vii.2008, Zi-Wei YIN leg. (SNUC). **Paratypes**: 17 ♂♂, 22 ♀ ♀, same label data as the holotype (SNUC); 4 ♂♂, 2 ♀♀, **CHINA**: Ningxia A. R., Guyuan City, Jingyuan County, Fengtai Forestry Station, alt. 2,400 m, 26.vi.2008, Wen-Xuan BI leg. (SNUC); 1 ♂, 1 ♀, **CHINA**: Qinghai Prov., Xining City, Huzhu County, Beishan, alt. 2,450 m, 28.vii.2004, TANG, HU & ZHU leg. (SNUC); 23 ♂♂, 16 ♀♀, **CHINA**: Qinghai Prov., Xining City, Huzhu County, Beishan, alt. 2,750 m, 29.vii.2004, TANG, HU & ZHU leg. (SNUC); 14 ♂♂, 62 ♀♀, **CHINA**: Qinghai Prov., Yunning Si (Lamasery), 2,890 m, 36°45.6'N, 102°10.6'E, 1.–16.vii.2005, J. Hájek, D. Král & J. Růžička leg. (NMP, cSch).

#### Description.

Measurements of males (holotype): BL 3.67–3.73 (3.67); FL 3.06–3.17 (3.06) mm;PL 0.89–0.95 (0.89); EL 1.45–1.50 (1.50); SEL 0.11–0.17 (0.11); HW 0.83–0.89 (0.83); PW 1.44–1.46 (1.45); EW 1.61–1.67 (1.61); relative length of antennomeres I–XI: 21: 15: 15: 10: 14: 12: 13: 13: 13: 13: 25. Measurements of females: BL 4.17–4.45; FL 3.34–3.50; PL 0.99–1.01; EL 1.66–1.68; SEL 0.21–0.23; HW 0.89–0.95; PW 1.50–1.61; EW 1.83–1.89; relative length of antennomeres I–XI: 25: 15: 16: 9: 15: 14: 14: 14: 13: 13: 25.

**Figure 2.**
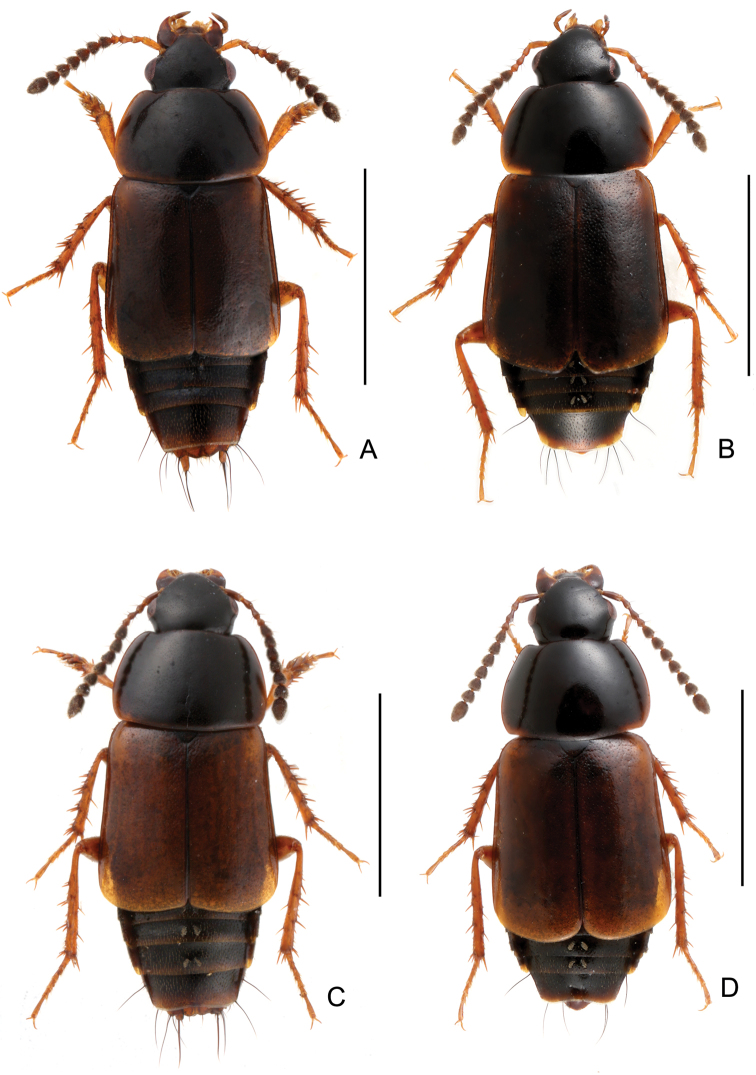
Habitus. **A** *Tachinus coronatus*, male holotype **B** *Tachinus coronatus*, female **C** *Tachinus hercules*, male holotype **D** *Tachinus hercules*, female. Scales: 2 mm.

Body (Figs 2A, 2B) dark brown to black; head and disc of pronotum black; elytra brown; mouthparts, basal four antennomeres, lateral margins of pronotum, small humeral spots on elytra, posterior margin of abdominal tergites and legs reddish brown.

Head slightly transverse, HW: PW = 0.55–0.62 (0.57); surface with fine microsculpture consisting of irregular striae, punctation moderately fine and sparse. Antennomeres X shorter than wide.

Pronotum: PL: PW = 0.61–0.67 (0.61); sides widest near base; surface with microsculpture similar to that of head, punctation slightly finer than that of head.

Elytra: EL: PL = 1.53–1.69 (1.69), EL: EW = 0.87–0.93 (0.93), EW: PW = 1.10–1.16 in males; EL: PL = 1.64–1.70; EL: EW = 0.88–0.92; EW: PW = 1.14–1.26 in females; posterior margin slightly rounded. Surface with coarser punctation than on pronotum, microsculpture distinct, consisting of irregular transverse meshes.

Male. Sternite VII (Figs 9C, 10C) similar to that of *Tachinus hujiayaoi*, with moderately deep triangular emargination at posterior margin, apical margin bent ventrad, with broad area of coarse granules. Tergite VIII as in Fig. 11C, with four almost fused lobes, forming a sinuate apical margin. Sternite VIII as in Fig. 13C. Median lobe of aedeagus (Fig. 15C) short and broad, distinctly projecting beyond apices of parameres, apical portion of median lobe with projection of unique shape. Parameres short, directed latero-apicad.

Female. Pronotum and elytra with microsculpture slightly more distinct. Elytra distinctly longer than in male, apical margin of elytra broadly rounded, without distinct projection near sutural angle. Tergite VIII (Fig. 18C) and sternite VIII (Fig. 20C) similar to those of *Tachinus hujiayaoi*.

#### Etymology.

The specific name (Latin adjective), meaning “crowned”, refers to the unique apical projection of the median lobe of the aedeagus.

#### Remarks.

Males of this can be separated from those of the other species by the unique projection on the apex of the median lobe of the aedeagus. Females are distinguished from those of all other species except *Tachinus hujiayaoi* by the shape of the apical margin of tergite VIII with two pairs of long setae.

### Tachinus (s. str.) hercules

Feng, Li & Schülke sp. n.

http://zoobank.org/C7AF0BFD-AD27-40BE-AC4A-CCF9A9AE7FB2

http://species-id.net/wiki/Tachinus_hercules

Figs 2C , 2D , 9D , 10D , 11D , 13D , 15D , 18D , 20D , 21D

#### Type locality.

China, Sichuan Province, Songpan, Huanglongsi.

#### Type material.

**Holotype**: ♂, **CHINA**: Sichuan Prov., Aba A. R., Songpan County, Huanglongsi, 24.vii.2001, LI & ZHAO leg. (SNUC). **Paratypes**: 25 ♂♂, 29 ♀♀, same label data as holotype (SNUC); 7 ♂♂, 10 ♀♀, **CHINA**: Sichuan Prov., pass between Songpan and Nanping, E–side, 3,450–3,500 m, 21.vi.2002, S. Murzin & I. Shokhin leg. (cSch); ♂, ♀, **CHINA**: Sichuan Prov., Zhangla env., 4,200–4,700 m, 9–11.vii.1991, J. Kaláb leg. (NHMB, cSch) [labelled as paratypes of *Tachinus szechuanensis* Campbell in litteris]; ♂, **CHINA**: Sichuan Prov., Songpan, 2,000 m, 32°30'N, 103°40'E, 13–17.vii.1990, J. Kolibáč leg. (NHMB); 3 ♂♂, 1 ♀, **CHINA**: Sichuan Prov., Min Shan, 33°10'N, 103°50'E, 2,500–4,500 m, 14–16.vii.1990, J. Kolibáč leg. (NHMB); 5 ♂♂, 1 ♀, **CHINA**: Sichuan Prov., pass between Pingwu and Jiuzhaigou, 3,000 m, 10–15.vii.2005, V. Patrikeev leg. (cSch); 1 ♂, 3 ♀♀, **CHINA**: N–Sichuan [CH12–23] Min Shan, 17 km NE Songpan, E pass, 32°44'23"N, 103°44'31"E, 3920 m, N–slope with *Rhododendron* below rocks, litter, moss, and grass roots sifted, 10.viii.2012, leg. M. Schülke (cSch); 3 ♂♂, 3 ♀♀, **CHINA**: N–Sichuan [CH12–24] pass 35 km NNW Songpan, 32°55'32"N, 103°25'56"E, 3600 m, moist N–slope with Salix and other shrubs, litter, grass roots, and moss sifted, 11.viii.2012, leg. M. Schülke (cSch); ♀, **CHINA**: N–Sichuan [CH12–19] 47 km N Songpan, road S 301 km 118, N Gongangling pass, 33°03'15"N, 103°43'36"E, 3390 m, spruce forest with shrubs, litter, moss, and mushrooms sifted, 9.viii.2012, leg. M. Schülke (cSch); ♂♀, **CHINA**: [19] N–Sichuan N Songpan, 33°03'15"N, 103°43'36"E, 3390 m, spruce forest, sifted, 9.viii.2012, V. Assing (cAss); ♀, **CHINA**: [22] N–Sichuan pass ENE Songpan, 4080 m, 32°44'54"N, 103°43'43"E, sifted. 10.viii.2012, V. Assing (cAss).

#### Description.

Measurements of males (holotype): BL 3.61–4.00 (4.00); FL 2.84–3.11 (3.11); PL 0.89–1.00 (1.00); EL 1.45–1.61 (1.61); SEL 0.06–0.11 (0.11); HW 0.88–0.90 (0.89); PW 1.45–1.56 (1.56); EW 1.61–1.78 (1.78); relative length of antennomeres I–XI: 23: 15: 15: 10: 17: 14: 16: 15: 15: 14: 29. Measurements of females: BL 3.95–4.39; FL 3.39–3.61 mm; PL 0.99–1.01; EL 1.56–1.73; SEL 0.16–0.18; HW 0.94–0.96; PW 1.50–1.56; EW 1.82–1.84; relative length of antennomeres I–XI: 23: 15: 15: 10: 14: 14: 14: 13: 14: 15: 28.

Body (Figs 2C, 2D) dark brown to black, head black, mouthparts, basal four antennomeres, elytra, posterior margin of each abdominal tergite and legs reddish brown.

Head shorter than wide and narrower than pronotum, HW: PW = 0.56–0.64 (0.57), disc of head with dense microsculpture consisting of irregular striae, basal part with microsculpture consisting of transverse meshes, punctation fine and sparse. Antennae moderately short, antennomeres X shorter than wide.

Pronotum: PL: PW = 0.57–0.69 (0.64), widest at basal third; surface with punctation slightly more distinct than that of head, microsculpture consisting of irregular transverse striae.

Elytra elongate: EL: PL = 1.45–1.81 (1.60), EL: EW = 0.81–1.00 (0.90), EW: PW = 1.03–1.23 (1.14) in males; EL: PL = 1.54–1.75, EL: EW = 0.85–0.95, EW: PW = 1.17–1.23 in females. Microsculpture consisting of fine, short meshes, punctation coarser and denser than that of head and pronotum.

Surface of abdomen evenly pubescent and punctate, microsculpture consisting of transverse lines.

Male. Sternite VII (Figs 9D, 10D) with posterior margin broadly and deeply emarginate, apical margin bent ventrad, with moderately broad area of coarse granules. Tergite VIII (Fig. 11D) with four short lobes, median lobes slightly longer than lateral ones. Sternite VIII as in Fig. 13D. Median lobe of aedeagus (Fig. 15D) broad and with two apical projections, parameres long and directed latero-apicad.

Female. Antennomeres X only slightly shorter than wide (length: width = 0.93). Pronotum with microsculpture more distinct and denser than in male. Elytra slightly longer than in male; apical margin broadly rounded, with microsculpture more distinct, denser and with shorter meshes than in male. Tergite VIII (Fig. 18D) with lobes almost fused, apical margin of median lobe broadly and triangularly projecting beyond lateral lobes; lateral lobes each with one long seta. Sternite VIII (Fig. 20D) with six lobes, fimbriate median lobes nearly fused, with shallow emargination at middle, sublateral lobes as long as median ones.

#### Etymology.

The species is named after the Greek divine hero Heracles, whose Roman name is Hercules.

#### Remarks.

Males of the new species are similar to those of *Tachinus parahercules* externally, and can be separated only by aedeagal characters, they differ from the other species by the shape of the aedeagal median lobe (two apical projections, long parameres directed lateroapically). Females are distinguished by the shape of the apical margin of tergite VIII, from females of the similar *Tachinus hujiayaoi* and *Tachinus coronatus* by the presence of a single pair of long setae on the lateral lobes of tergite VIII.

### Tachinus (s. str.) hujiayaoi

Feng, Li & Schülke sp. n.

http://zoobank.org/E6D5EFCE-5897-4424-B265-3127A284CF51

http://species-id.net/wiki/Tachinus_hujiayaoi

Figs 3A , 3B , 9E , 10E , 11E , 13E , 15E , 18E , 20E , 21E

#### Type locality.

China, Shaanxi Province, Taibai Mountain.

#### Type material.

**Holotype**: ♂, **CHINA**: Shaanxi Prov., Baoji City, Taibai County, Mt. Taibai, alt. 2,750–3,300 m, 12.vii.2004, HU, TANG & ZHU leg. (SNUC). **Paratypes**: 56 ♂♂, 38 ♀♀, same label data as holotype (SNUC); 11 ♂♂, 17 ♀♀, **CHINA**: Shaanxi Prov., Baoji City, Taibai County, Mt. Taibai, alt. 2,750–3,300 m, 12.vii.2004, HU & TANG leg. (SNUC); 22 ♂♂, 12 ♀♀, **CHINA**: Shaanxi Prov., Qinling, 33°51'N, 108°47'E, mountain W pass at autoroute km 70, 47 km S Xi'an, 2,300–2,500 m, 26–30.viii.1995, A. Pütz, M. Schülke leg. (cPüt, cSch); 1 ♀, **CHINA**: Shaanxi Prov., Qinling, 33°51'N, 108°47'E, mountain W pass at autoroute km 70, 47 km S Xi'an, 2,500–2,600 m, 26–29.viii.1995, Wrase leg. (cSch); 1 ♀, **CHINA**: S–Shaanxi, Qinling, range W pass on rd. Xi'an–Shagoujie, 45 km SSW Xi'an, 33°52'N, 108°46'E, 2,600 m, 25.vii.2001, A. Smetana leg. (cSme).

#### Description.

Measurements of males (holotype): BL 4.23–4.61 (4.61); FL 3.11–3.22 (3.22);PL 0.89–0.95 (0.95); EL 1.33–1.45 (1.45); SEL 0.08–0.11 (0.11); HW 0.82–0.84 (0.83); PW 1.39–1.45 (1.45); EW 1.56–1.61 (1.61); relative length of antennomeres I–XI: 23: 17: 17: 8: 13: 12: 12: 13: 13: 14: 27. Measurements of females: BL 4.33–4.45; FL 3.45–3.50;PL 0.89–0.95; EL 1.61–1.72; SEL 0.22–0.28; HW 0.88–1.00; PW 1.45–1.50; EW 1.78–1.83; relative length of antennomeres I–XI: 24: 18: 16: 11: 15: 13: 13: 13: 12: 13: 30.

**Figure 3.**
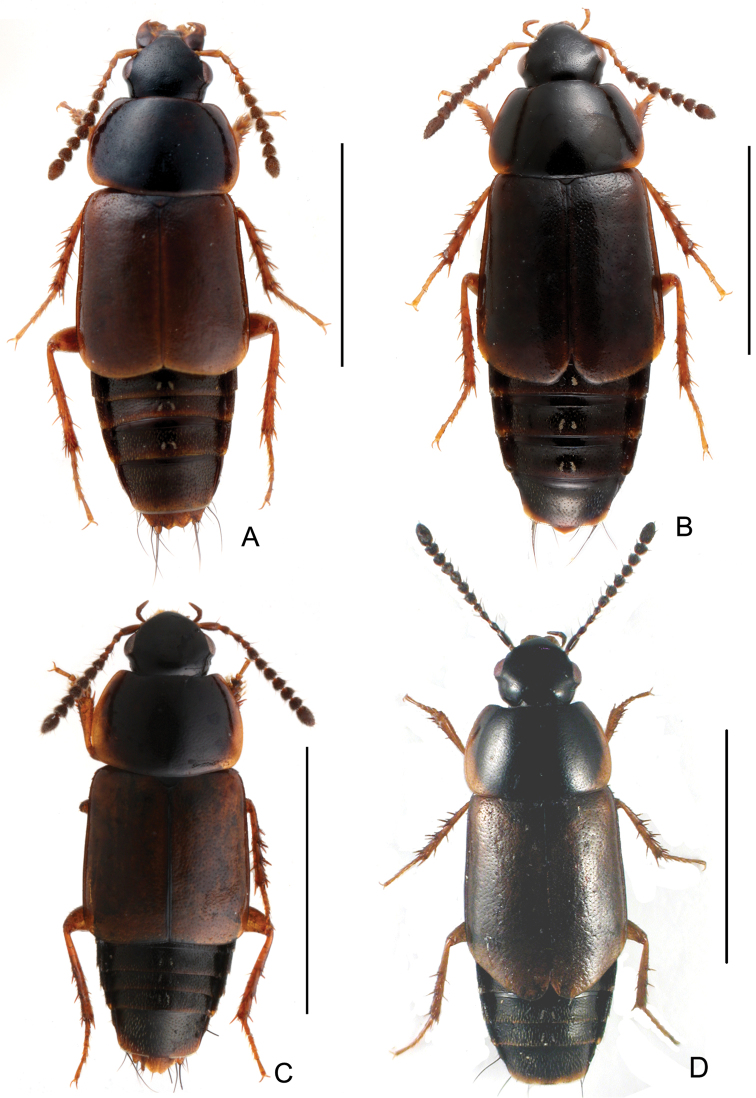
Habitus. **A** *Tachinus hujiayaoi*, male holotype **B** *Tachinus hujiayaoi*, female **C** *Tachinus jiuzhaigouensis*, male holotype **D** *Tachinus jiuzhaigouensis*, female. Scales: 2 mm.

Body (Figs 3A, 3B) dark brown to black, disc of elytra brown, mouthparts, basal four antennomeres, lateral margins of pronotum, posterior margin of elytra, abdominal tergites and legs reddish brown.

Head transverse, HW: PW = 0.57–0.60 (0.57) in males, HW: PW = 0.59–0.69 in females;disc with microsculpture consisting of irregular striae, posterior portion with microsculpture consisting of transverse meshes, punctation fine and sparse. Antennae moderately short, antennomeres X slightly shorter than wide.

Pronotum: PL: PW = 0.59–0.68 (0.66), punctation similar to that of head, microsculpture consisting of irregular transverse meshes.

Elytra: EL: EW = 0.83–0.93 (0.90), EL: PL = 1.40–1.63 (1.53), EW: PW = 1.08–1.16 (1.11) in males; EL: EW = 0.88–0.97, EL: PL = 1.69–1.93, EW: PW = 1.19–1.26 in females; apical margin broadly rounded, punctation coarser than that of head and pronotum, microsculpture more distinct than on head and pronotum, consisting of shorter transverse meshes.

Abdomen with fine microsculpture consisting of transverse lines.

Male. Sternite VII (Figs 9E, 10E) triangularly emarginate at posterior margin, apical margin bent ventrad, with moderately broad area of coarse granules. Tergite VIII as in Fig. 11E. Sternite VIII as in Fig. 13E, all four lobes nearly fused, forming a sinuate apical margin. Median lobe of aedeagus (Fig. 15E) apically distinctly projecting beyond apices of parameres, apical part with characteristic projections.

Female. Elytra slightly longer than in male, inner part of posterior margin weakly produced, forming a broadly obtuse angle. Tergite VIII (Fig. 18E) with lobes almost fused, apical margin of median lobe broadly and triangularly projecting, as long as lateral lobes; lateral lobes each with one pair of long setae. Sternite VIII (Fig. 20E) with six lobes, fimbriate median lobes nearly fused, with small and shallow emargination, sublateral lobes slightly longer than fimbriate median lobes.

#### Etymology.

The species is named after Jia-Yao Hu (Shanghai), a colleague and friend specializing in Paederinae and Quediina.

#### Remarks.

The new species is most similar to *Tachinus coronatus* externally, and can be separated only by aedeagal characters. Males of *Tachinus hujiayaoi* can be differed from those of the other species by the unique shape of the apical projections of the aedeagal median lobe. Females are distinguished from those of all other species except *Tachinus coronatus* by the shape of the apical margin of tergite VIII (lateral lobes each with one pair of long setae).

### Tachinus (s. str.) jiuzhaigouensis

Feng, Li & Schülke sp. n.

http://zoobank.org/22962962-16D0-4562-A5DE-B4B3C8848FE1

http://species-id.net/wiki/Tachinus_jiuzhaigouensis

Figs 3C , 3D , 9F , 10F , 11F , 13F , 15F , 18F , 20F , 21F

#### Type locality.

China, Sichuan, Aba A. R., Nanping, Jiuzhaigou Natural Reserve.

#### Type material.

**Holotype**: ♂, **CHINA**: Sichuan Prov., Aba A. R., Nanping County, Jiuzhaigou, 27.vii.2001, LI & ZHAO leg. (SNUC). **Paratypes**: ♂, same label data as holotype (SNUC); ♂, **CHINA**: Sichuan Prov., Jiuzhaigou Natural Reserve, 12–17.vi.2000, leg. E. Kučera (cFel); 4 ♂♂, 6 ♀♀, **CHINA**: Sichuan, Jiuzhaigou, 15.vi.–18.vi.2011 leg. Kučera (cSch).

#### Description.

Measurements of holotype: BL 3.61 mm; FL 2.61 mm;PL 0.78 mm; EL 1.17 mm; SEL 0.06; HW 0.72 mm; PW 1.22 mm; EW 1.33 mm; relative length of antennomeres I–XI: 20: 10: 12: 7: 8: 10: 10: 10: 9: 10: 21. Measurements of females: BL 3.57–3.81; FL 2.82–3.08; PL 0.79–0.83; EL 1.29–1.44; SEL 0.20–0.30; HW 0.73–0.74; PW 1.22–1.26; EW 1.34–1.39; relative length of antennomeres I–XI: 24: 14: 16: 10: 14: 14.5: 14: 14: 13: 12.5: 26.

Body (Fig. 3C, 3D) brown to black; head and pronotal disc black; lateral margins of pronotum yellow; elytra, posterior margin of each abdominal tergite and legs reddish brown; mouthparts and basal antennomeres black or dark brownish.

Head slightly transverse, HW: PW = 0.58–0.60, punctation fine and sparse, microsculpture consisting of irregular striae on disc, more transverse in posterior portion. Antennomeres X shorter than wide.

Pronotum: PL: PW = 0.64–0.66; surface with fine and sparse punctation, microsculpture indistinct, reduced to irregular transverse striae.

Elytra: EL: PL = 1.50, EL: EW = 0.88, EW: PW = 1.09 in male; EL: PL = 1.61–1.74; EL: EW = 0.93–1.04; EW: PW = 1.08–1.11 in females; punctation shallow, coarser than that of pronotum.

Male. Surface of elytra with microsculpture similar to that of pronotum, often almost completely reduced. Sternite VII (Figs 9F, 10F) as in *Tachinus lohsei*, apical margin of emargination more rounded, hand-shaped projection more slender, in posterior view apical margin bent dorsad. Tergite VIII as in Fig. 11F. Sternite VIII as in Fig. 13F. Median lobe of aedeagus (Fig. 15F) shorter and slightly broader than parameres. Parameres in lateral view straight, not curved ventrad in apical half, apex of parameres angular in ventral view.

Female. Elytra distinctly longer than in male (Fig. 3D), inner part of posterior margin produced, forming a distinct angle. Surface with distinct microsculpture composed of irregular transverse striae in the anterior portion and of irregular meshes in posterior portion. Abdominal tergite VIII (Fig. 18F) trilobed, similar to that of *Tachinus lohsei*, median lobe at least as long as lateral ones, lateral lobes broad and each with one pair of long setae. Sternite VIII (Fig. 20F) with six lobes, fimbriate median lobes separated by deep and narrow emargination.

#### Etymology.

The specific name (adjective) is derived from “Jiuzhaigou”, the type locality of this species.

#### Remarks.

*Tachinus jiuzhaigouensis* can be easily separated from the other species of the group by its small size, the broadly yellow lateral margins of the pronotum and the primary and secondary sexual characters. The species is very similar to *Tachinus lohsei*, but the male is distinguished by the more angular apical part of the parameres, the female by the different shape of the median lobe of tergite VIII.

### Tachinus (s. str.) linzhiensis

Feng & Li sp. n.

http://zoobank.org/821FEE94-B530-45C9-8706-D51D0E6DD32C

http://species-id.net/wiki/Tachinus_linzhiensis

Figs 4A , 4B , 9G , 10G , 11G , 13G , 16A , 18G , 20G , 21G

#### Type locality.

China, Tibet A. R., Linzhi County, Basongcuo.

#### Type material.

**Holotype**: ♂, **CHINA**: Tibet A. R., Linzhi County, Basongcuo, alt. 3,465 m, 9.viii.2004, Li-Zhen LI leg. (SNUC). **Paratypes**: 11 ♂♂, 5 ♀♀, same label data as holotype (SNUC).

#### Description.

Measurements of males (holotype):BL 3.89–4.17 (3.89); FL 2.95–3.17 (2.95); PL 0.94–0.96 (0.95); EL 1.45–1.56 (1.56); SEL 0.06–0.08 (0.06); HW 0.83–0.89 (0.89); PW 1.39–1.45 (1.39); EW 1.56–1.61 (1.56); relative length of antennomeres I–XI: 21: 13: 14: 10: 15: 12: 13: 12: 13: 12: 27. Measurements of females: BL 4.84–5.12; FL 3.61–3.73; PL 0.95–1.00; EL 1.61–1.67; SEL 0.11–0.14; HW 0.88–0.90; PW 1.49–1.51; EW 1.72–1.78; relative length of antennomeres I–XI: 20: 14: 15: 10: 14: 14: 14: 14: 14: 14: 24.

**Figure 4.**
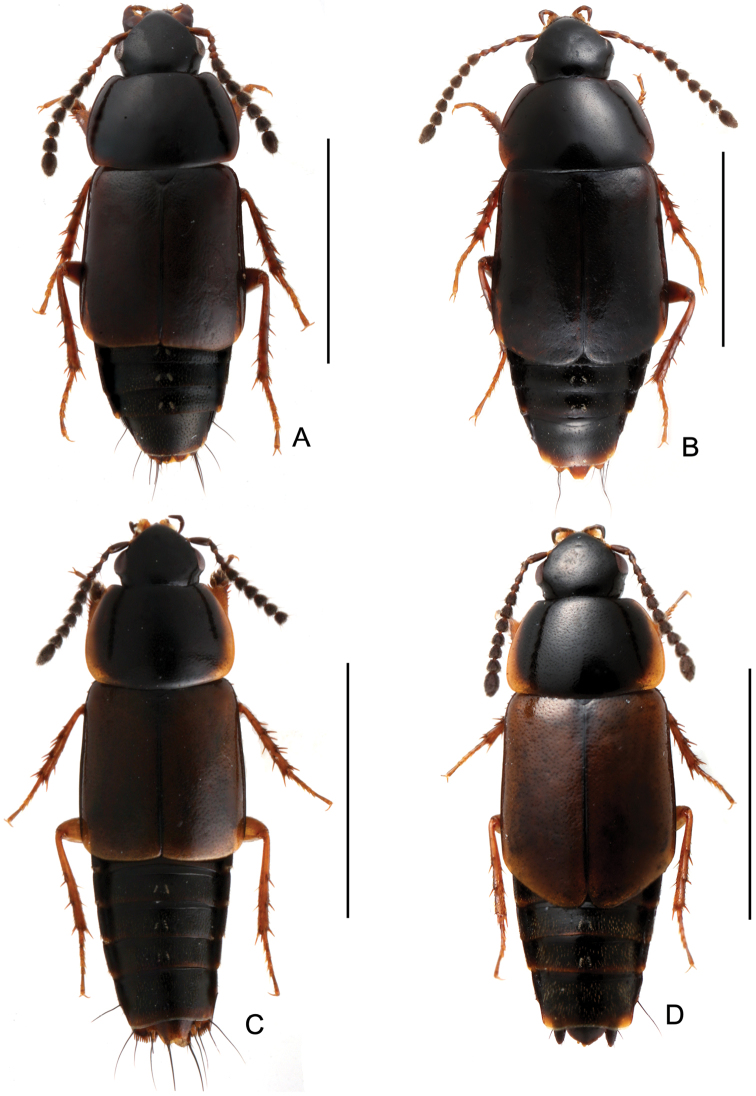
Habitus. **A** *Tachinus linzhiensis*, male holotype **B** *Tachinus linzhiensis*, female **C** *Tachinus lohsei*, male **D** *Tachinus lohsei*, female. Scales: 2 mm.

Body (Figs 4A, 4B) black to piceous; mouthparts, antennomeres I and IV, elytra and legs reddish brown.

Head slightly transverse, HW: PW = 0.57–0.64 (0.64). Surface of head with distinct microsculpture and fine and sparse punctation. Antennae moderately short, antennomeres X slightly transverse or as long as wide.

Pronotum: PL: PW = 0.63–0.69 (0.68), surface with microsculpture consisting of transverse irregular meshes, punctation finer than that of head.

Elytra short, EL: EW = 0.90–1.00 (1.00), EL: PL = 1.51–1.66 (1.64), EW: PW = 1.08–1.16 (0.12) in males; EL: EW = 0.90–0.97, EL: PL = 1.61–1.76, EW: PW = 1.14–1.19 in females; with microsculpture consisting of transverse meshes, more coarsely punctate than head and pronotum.

Surface of abdomen with dense microsculpture consisting of transverse lines, punctation similar to that of elytra.

Male. Sternite VII (Figs 9G, 10G) only shallowly emarginate at posterior margin, apical margin bent ventrad, with broad area of coarse granules. Tergite VIII (Fig. 11G) with four short lobes, median lobes broader and slightly longer than lateral ones. Sternite VIII as in Fig. 13G. Aedeagus (Fig. 16A) with parameres much longer than median lobe; parameres directed apicad, slightly curved medially. Parameres projecting beyond apex of median lobe by one quarter length of median lobe.

Female. Elytra only slightly longer than in male, apical margin broadly rounded. Tergite VIII (Fig. 18G) long and broad, distinctly trilobed, lateral lobes shorter than median lobe, apical margin of median lobe slightly emarginate at middle; lateral lobes each with one pair of long setae. Sternite VIII (Fig. 20G) with fimbriate median lobes completely fused.

#### Etymology.

The specific name (adjective) is derived from “Linzhi”, the type locality of this species.

#### Remarks.

Males can be separated from those of the other species of the group by the apically strongly elongate aedeagal parameres. Females are distinguished from those of all other species except *Tachinus paralinzhiensis* by the shape of tergite VIII, from those of *Tachinus paralinzhiensis* by the totally fused fimbriate lobe of sternite VIII.

### Tachinus (s. str.) lohsei

Ullrich

http://species-id.net/wiki/Tachinus_lohsei

Figs 4C , 4D , 8C , 8D , 9H , 10H , 11H , 13H , 16B , 18H , 20H , 21H

Tachinus lohsei (*s. str.*) Ullrich, 1975: 277.

#### Type locality.

China, Sichuan Province, Wenchuan, Yingxiu.

#### Type material examined.

**Holotype** ♂ (Fig. 8C, 8D): **CHINA**: “Wassuland Bzk. Sankiangkou W Szechuan, China Coll. H. Becker / Ninto Shan 2400 m 4.VII.'34 / Holotypus *Tachinus lohsei* n. sp. des. W.G. Ullrich 1974, ‘3014' [red] / *Tachinus lohsei* n. sp. ♂ W.G. Ullrich det. 1974, ‘3014'” (MHNG).

#### Other material examined.

6 ♂♂, 5 ♀♀, CHINA: Sichuan Prov., Ganzi A. R., Luding County, Hailuogou, Ganheba, alt. 3,300 m, 26.vi.2009, Li-Zhen LI leg. (SNUC); 1 ♂, 1 ♀, CHINA: Sichuan Prov. Ganzi A. R., Luding County, Hailuogou, Gaohaizi, alt. 2,780 m, 28.vi.2009, Li-Zhen LI leg. (SNUC); 4 ♂♂, 1 ♀, CHINA: Sichuan Prov. Ganzi A. R., Luding County, Hailuogou, Shengtaizhan, alt. 3,000 m, 25.vi.2009, Li-Zhen LI leg. (SNUC); 1 ♀, CHINA: Sichuan Prov., Gongga Shan mts., NNE–slope, 4,000 m, 29°53'N, 102°01'E, 9.–11.vii.1994, D. Král & J. Farkač leg. (NHMB); ♂, ♀, CHINA: Sichuan Prov., Gongga Shan–Hailuogou, 2,900–3,200 m, 29°35'N, 102°00'E, 3–8.vii.1994, D. Král & J. Farkač leg. (cSme, cSch); 1 ♀, CHINA: Sichuan Prov., Gongga Shan, above Camp 2, 2,800 m, 25.vii.1994, A. Smetana leg. (cSme); ♂, CHINA: Sichuan Prov., Gongga Shan, above Camp 2, 2,850 m, 26.vii.1994, A. Smetana leg. (cSme); 1 ♂, 1 ♀, CHINA: Sichuan Prov., Gongga Shan, above Camp 2, 2,800 m, 29°35'N, 102°00'E, 5.vii.1998, A. Smetana leg. (cSme, cSch); 2 ♂♂, 1 ♀, CHINA: Sichuan Prov., Gongga Shan, above Camp 3, 3,200 m, 29°35'N, 102°00'E, 7.vii.1998, A. Smetana leg. (cSme, cSch); 5 ♂♂, 2 ♀♀, CHINA: Sichuan Prov., Gongga Shan, above Camp 3, 3,050 m, 29°35'N, 102°00'E, 6.vii.1998, A. Smetana leg. (cSme, cSch); 1 ♂, 2 ♀♀, CHINA:  Sichuan, Gongga Shan, Hailuogou, above Camp 3, 3200 m, 7.VII. 1996 29°35'N, 102°00'E / collected by J. Farkač, P. Kabátek and A. Smetana (NHMB, cSch); 1 ♀, CHINA:  Sichuan, Gongga Shan, Hailuogou, above Camp 3, 29°35'N, 102°00'E, 2800–3200 m, 6.–8.VII.1998 J. Farkač / 1998 China Expedition J. Farkač, D. Král, J. Schneider & A. Smetana (NHMB); 1 ♀, CHINA:  Sichuan, Gongga Shan, Hailuogou, above Camp 2, 29°35'N, 102°00'E, 2600–2750 m, 3.–6.VII.1998 J. Schneider / 1998 China Expedition J. Farkač, D. Král, J. Schneider & A. Smetana (NHMB); 1 ♂, 1 ♀, CHINA: Sichuan, Gongga Shan, Hailuogou, above Camp 2, 29°35'N, 102°00'E, 2600–2750 m, 3.–6.VII.1998 J. Farkač (NHMB).

#### Description.

Measurements of males: BL 4.06–4.17; FL 2.84–3.22; PL 0.83–0.84; EL 1.31–1.33; SEL 0.03–0.06; HW 0.72–0.73; PW 1.17–1.28; EW 1.33–1.39; relative length of antennomeres I–XI: 22: 14: 14: 8: 10: 10: 11: 10: 10: 12: 22. Measurements of females: BL 4.17–4.23; FL 2.95–3.11; PL 0.82–0.83; EL 1.50–1.56; SEL 0.11–0.17; HW 0.72–0.78; PW 1.27–1.29; EW 1.39–1.45; relative length of antennomeres I–XI: 21: 14: 15: 12: 13: 13: 14: 12: 12: 11: 25.

Body (Figs 4C, 4D) dark brown to black, head and pronotal disc black, lateral margins of pronotum broadly yellowish brown, elytral disc brown, mouthparts, basal four antennomeres, posterior margin of elytra, abdominal tergites and legs reddish brown.

Head slightly transverse, HW: PW = 0.55–0.62, surface with fine and sparse punctation, microsculpture consisting of discontinuous striae. Antennomeres X slightly shorter than wide in male, but more distinctly shorter than wide in female.

Pronotum: PL: PW = 0.64–0.72, punctation slightly coarser than that of head.

Elytra: EL: PL = 1.56–1.62, EL: EW = 0.94–1.00, EW: PW = 1.04–1.19 in males; EL: PL = 1.79–1.90, EL: EW = 1.03–1.08, EW: PW = 1.08–1.14 in females; apical margin broadly rounded. Surface of elytra with punctation coarser than that of pronotum.

Abdomen with denser and finer punctation than elytra.

Male. Surface of pronotum with weak microsculpture, elytra only with traces of microsculpture. Sternite VII (Figs 9H, 10H) with large basal projection and deep median triangular emargination, coarse granules arranged as inverted “V” in anterior portion of impression, each side of impression with a hand-shaped projection, lateral sides of apical margin with long comb-like setae; apical margin bent dorsad when viewed from behind. Tergite VIII (Fig. 11H) with four lobes, median lobes with small, narrowly rounded emargination between them, and longer than lateral ones. Sternite VIII (Fig. 13H) with four distinct lobes, median lobes longer than lateral ones and separated by a deep, bell-shaped emargination. Aedeagus (Fig. 16B) with median lobe shorter than the parameres, parameres wide and elongate, sides gradually narrowed from apical half to rounded apices; apical third of lateral lobes slightly curved ventrad in lateral view.

Female. Pronotum with microsculpture more distinct, consisting of sinuate transverse striae. Elytra distinctly longer than in male, inner part of posterior margin slightly produced, forming an obtuse angle, microsculpture much more distinct, consisting of short transverse to isodiametric meshes. Tergite VIII (Fig. 18H) distinctly trilobed, median lobe separated from lateral lobes by a deep suture, shorter than lateral ones; lateral lobes broad, each with one pair of long setae. Sternite VIII (Fig. 20H) with six lobes, fimbriate median lobes separated by deep and narrow emargination.

#### Remarks.

*Tachinus lohsei* can be separated from the other species of the group by its small size, the broadly yellow lateral margins of the pronotum, and the different primary and secondary sexual characters. The species is very similar to *Tachinus jiuzhaigouensis*, but the male is distinguished by the different, more rounded apical part of the parameres, the female by the different shape of the median lobe of tergite VIII.

### Tachinus (s. str.) maderi

Bernhauer

http://species-id.net/wiki/Tachinus_maderi

Figs 8A , 8B , 18I , 20I , 21I

Tachinus maderi (*s. str.*) Bernhauer, 1939: 156 f.
Tachinus maderi (*s. str.*) : Ullrich 1975: 278.

#### Type locality.

China, Sichuan Province, Kangding.

#### Type material examined.

**Holotype** ♀ (Fig. 8A, 8B): **CHINA**,“[old mounting plate] / Tatsienlu–Kiulung China Em. Reitter / Maderi Brnh. typus unic. *Tachinus* / *Tachinus Maderi* Brh. Typus unic. / Chicago NHMus. M. Bernhauer Collection / Lectotypus des. W.G. Ullrich 1973 5689 / *Tachinus maderi* Bernh. 1 ♀, W.G. Ullrich vid. 5689 / HOLOTYPUS– ♀ *Tachinus (Tachinus) maderi* Bernh., 1939 M. Schülke 2007 / *Tachinus (Tachinus) maderi* Bernhauer det. M. Schülke 2007”.

#### Description.

Measurements of female holotype: BL 4.63; FL 3.90; PL 1.07; EL 1.80; SEL 0.34; HW 0.93; PW 1.61; EW 1.78; relative length of antennomeres I–XI: 16: 9: 10: 6.5: 10: 8: 9: 9: 8.5: 8.5: 16.

Body (Fig. 8A) dark brown to black; margins of elytra, mouthparts, basal four antennomeres and legs paler brown.

Head slightly transverse, HW: PW = 0.58, surface almost without punctation, microsculpture consisting of irregular striae. Antennomeres X only slightly shorter than wide.

Pronotum: PL: PW = 0.67; surface only with very fine and sparse punctation, microsculpture consisting of irregular, mostly transverse striae.

Elytra elongate, EL: EW = 1.01, EL: PL = 1.68, EW: PW = 1.11; inner part of posterior margin distinctly produced, forming a distinct angle. Surface of elytra with uneven and sparse punctation, microsculpture distinct, consisting of fine rhomboid or somewhat transverse meshes.

Abdomen with microsculpture consisting of transverse striae, punctation moderately coarse and dense.

Male. Unknown.

Female. Posterior margin of tergite VIII (Fig. 18I) sinuate, without obvious median lobes, but with short acute lobes at lateral angles, each with one pair of long setae. Sternite VIII (Fig. 20I) with fimbriate median lobes nearly fused; sublateral lobes as long as median ones.

#### Remarks.

Females of this species are distinguished from those of all other species by the slender habitus with exceptionally elongate elytra; from all species except the similar *Tachinus armatus* and *Tachinus parahercules* by the shape of tergite VIII.

### Tachinus (s. str.) maderianus

Feng & Li sp. n.

http://zoobank.org/C486F05A-C320-4BA1-8709-C268C9CD3D8B

http://species-id.net/wiki/Tachinus_maderianus

Figs 5A , 5B , 9I , 10I , 11I , 13I , 16C , 19A , 20J , 21J

#### Type locality.

China, Sichuan, Jiulong, Wahuishan Mountain.

#### Type material.

**Holotype**: ♂, **CHINA**: Sichuan Prov., Ganzi A. R., Jiulong County, Wahuishan, alt. 3,900 m, 26.viii.2005, [collector unknown] (SNUC). **Paratypes**: 2 ♂♂, 4 ♀♀, same label data as holotype; 1 ♂, 2 ♀♀, **CHINA**: Sichuan Prov., Ganzi A. R., Jiulong County, Wuxuhai, alt. 3,700 m, 18.viii.2005, [collector unknown] (SNUC).

#### Description.

Measurements of males: BL 4.26–4.39; FL 3.34–3.39; PL 0.99–1.01; EL 1.55–1.57; SEL 0.11–0.17; HW 0.83–0.89; PW 1.44–1.46; EW 1.61–1.72; relative length of antennomeres I–XI: 24: 15: 15: 8: 15: 13: 13: 11: 12: 12: 25. Measurements of females: BL 4.67–4.73; FL 4.00–4.11; PL 0.99–1.01; EL 1.78–1.83; SEL 0.38–0.40; HW 0.88–0.90; PW 1.53–1.56; EW 1.88–1.90; relative length of antennomeres I–XI: 25: 15: 18: 10: 14: 14: 14: 14: 14: 14: 24.

**Figure 5.**
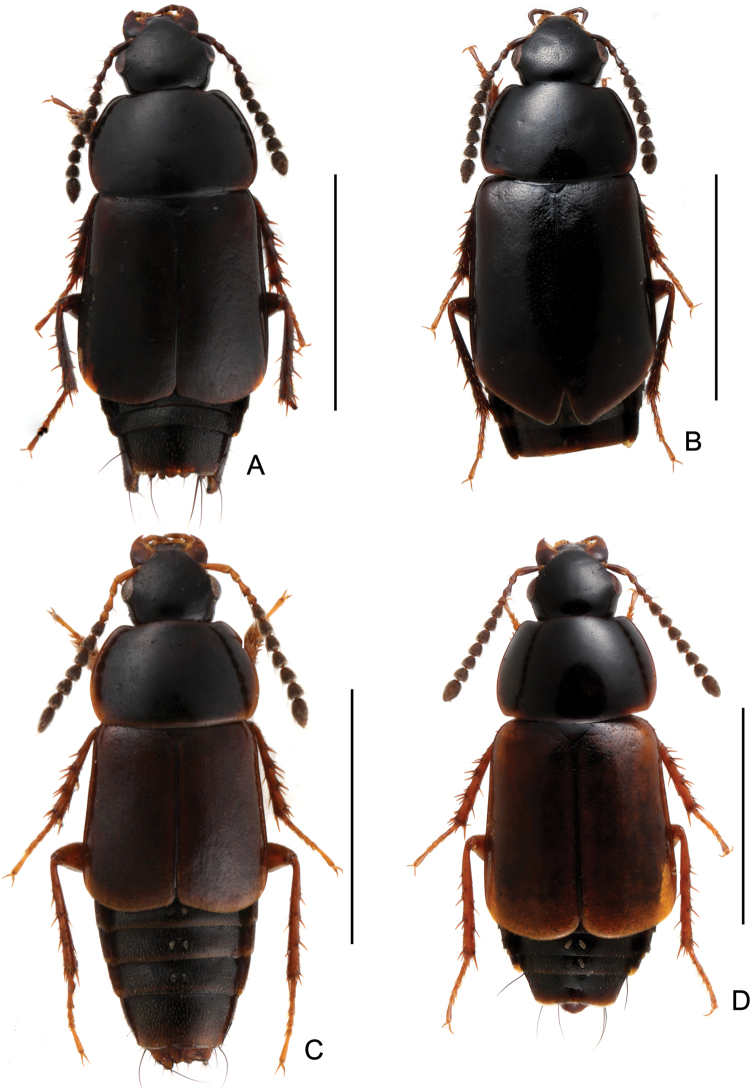
Habitus. **A** *Tachinus maderianus*, male holotype **B** *Tachinus maderianus*, female **C** *Tachinus mengdaensis*, male holotype **D** *Tachinus mengdaensis*, female. Scales: 2 mm.

Body (Figs 5A, 5B) dark brown to black; mouthparts, basal four antennomeres and legs reddish brown.

Head slightly transverse, HW: PW = 0.56–0.62, surface with fine punctation and dense microsculpture consisting of meshes. Antennomeres X distinctly shorter than wide in male, but slightly shorter than wide in female.

Pronotum: PL: PW = 0.68–0.70 (male), PL: PW = 0.63–0.66 (female); surface with punctation similar to that of head, microsculpture consisting of transverse striae.

Elytra: EL: PL = 1.53–1.59, EL: EW = 0.90–0.98, EW: PW = 1.10–1.19 in males; EL: PL = 1.76–1.85, EL: EW = 0.94–0.97, EW: PW = 1.21–1.24 in females; surface with coarser punctation than head and pronotum, microsculpture consisting of transverse meshes.

Abdomen with microsculpture consisting of transverse striae, punctation moderately coarse and dense.

Male. Posterior margin of elytra broadly rounded. Sternite VII (Figs 9I, 10I) with apical margin broadly and deeply emarginate, apical margin bent ventrad, with very broad area of coarse granules. Tergite VIII as in Fig. 11I, all four lobes nearly fused, forming a sinuate apical margin. Sternite VIII as in Fig. 13I. Median lobe of aedeagus (Fig. 16C) as wide as, and longer than parameres, parameres forming one apically truncate plate.

Female. Inner part of posterior margin of elytra distinctly produced, forming a distinct angle. Posterior margin of tergite VIII (Fig. 19A) without distinct lobes, lateral angles each with only one long seta. Sternite VIII (Fig. 20J) with fimbriate median lobes separated by a shallow emargination; sublateral lobes as long as median ones.

#### Etymology.

The specific name (adjective) is derived from the name of the Austrian entomologist Leopold Mader (1886–1961), referring to the similarity of this species to *Tachinus maderi*.

#### Remarks.

Males can be separated from those of other species by the shape of the very broadly bent portion of sternite VII, as well as by the shape of the parameres, and the apical projection of the median lobe of the aedeagus. Females are distinguished by the shape of the apical margin of tergite VIII.

### Tachinus (s. str.) mengdaensis

Feng, Li & Schülke sp. n.

http://zoobank.org/98CD1D38-3279-4E87-ABB4-A87607E0A7F0

http://species-id.net/wiki/Tachinus_mengdaensis

Figs 5C , 5D , 9J , 10J , 12A , 13J , 16D , 19B , 20K , 22A

#### Type locality.

China, Qinghai Province, Xining, Mengda Natural Reserve.

#### Type material.

**Holotype**: ♂, **CHINA**: Qinghai Prov., Xining City, Xunhua County, Mengda N. R., alt. 2,200–2,500 m, 24.vii.2004, HU, TANG & ZHU leg. (SNUC). **Paratypes**: 18 ♂♂, 27 ♀♀, same label data as the holotype (SNUC); 4 ♂♂, **CHINA**: Gansu Prov., Dagcanglhamo (= Langmusi), 34°04.6'N, 102°37.7'E, 3644 m, J. Hájek, D. Král & J. Růžička leg [date unknown]. (NMP, cSch); 3 ♂♂, 7 ♀♀, **CHINA**: Gansu Prov., Hue er Ge, 5 km SSW Luqu, 3,400 m, 13.vii.1994, A. Smetana leg. (cSme, cSch); ♂, ♀, **CHINA**: Gansu Prov., Xiahe env., 3,000–3,200 m, 28.vii.–3.viii.1993, W. Heinz leg. (cSme); ♀, **CHINA**: Sichuan Prov., S of Langmusi, forest, 3,400–3,500 m, 13–14.VII.1994, K.W. Anton leg. (cSme); 4 ♀♀, **CHINA**: Sichuan Prov., Langmusi, 3,600 m, 14.vii.1994, A. Smetana leg. (cSme, cSch); 5 ♂♂, 4 ♀♀, **CHINA**: Sichuan Prov., Langmusi, 3,500 m, 13.vii.1994, A. Smetana leg. (cSme, cSch).

#### Description.

Measurements of males (holotype): BL 4.23–4.34 (4.24); FL 2.84–2.95 (2.84);PL 0.88–0.90 (0.89); EL 1.28–1.33 (1.33); SEL 0.10–0.12 (0.12); HW 0.82–0.84 (0.83); PW 1.39–1.45 (1.45); EW 1.56–1.67 (1.56); relative length of antennomeres I–XI: 23: 15: 17: 10: 14: 16: 16: 16: 14: 14: 27. Measurements of females: BL 4.17–4.34; FL 3.34–3.50; PL 0.95–1.00; EL 1.45–1.50; SEL 0.16–0.18; HW 0.71–0.83; PW 1.39–1.50; EW 1.67–1.78; relative length antennomeres I–XI: 24: 14: 16: 10: 15: 14: 14: 15: 15: 16: 30.

Body (Figs 5C, 5D) reddish to dark brown; mouthparts, margins of pronotum and posterior margin of tergites reddish brown; basal four antennomeres and legs yellowish brown.

Head slightly transverse, HW: PW = 0.47–0.60 (0.57); surface with very fine and sparse punctation, sometimes invisible in the pronounced microsculpture; microsculpture consisting of irregular striae. Antennomeres X slightly shorter than wide.

Pronotum: PL: PW = 0.61–0.72 (0.61); surface with microsculpture consisting of transverse striae, punctation similar to that of head.

Elytra: EL: PL = 1.42–1.51 (1.49), EL: EW = 0.77–0.85 (0.85), EW: PW = 1.08–1.20 (1.08) in males; EL: PL = 1.45–1.58, EL: EW = 0.81–0.90, EW: PW = 1.11–1.28 in females; surface with more distinct punctation than head and pronotum, microsculpture consisting of transverse meshes.

Abdomen with fine punctation, microsculpture consisting of transverse waves.

Male. Posterior margin of sternite VII (Figs 9J, 10J) with broad triangular emargination medially, apical margin bent ventrad, with broad area of coarse granules. Tergite VIII as in Fig. 12A, all four lobes nearly fused, forming a sinuate apical margin. Sternite VIII as in Fig. 13J. Median lobe of aedeagus (Fig. 16D) broad and projecting beyond apices of parameres, apical portion of median lobe in lateral view with belt-shaped projection.

Female. Pronotum with microsculpture more distinct, forming more regular transverse meshes. Elytra distinctly longer than in male, apical margins broadly rounded. Tergite VIII (Fig. 19B) transverse, lobes nearly fused, posterior margin of median lobe broadly rounded, lateral lobes each with only one pair of long setae. Fimbriate median lobes of sternite VIII (Fig. 20K) separated by a shallow emargination, slightly shorter than sublateral lobes.

#### Etymology.

The specific name (adjective) is derived from “Mengda”, the type locality of this species.

#### Remarks.

This new species can be separated from the other species by the triangular emargination of the apical margin of the male sternite VII, the belt-like projection (lateral view) of the aedeagal median lobe, the shape of the aedeagal parameres, and the broadly rounded median lobe of the female tergite VIII.

### Tachinus (s. str.) oblongoelytratus

Feng & Li sp. n.

http://zoobank.org/30F34AF6-F3F5-4A8F-BAB8-0A94AECCF38E

http://species-id.net/wiki/Tachinus_oblongoelytratus

Figs 6A , 6B , 9K , 10K , 12B , 13K , 16E , 19C , 20L , 22B

#### Type locality.

China, Sichuan, Emeishan.

#### Type material.

**Holotype**: ♂, **CHINA**: Sichuan Prov. Emeishan City, Mt. Emei, 17.vii.2003, Li-Zhen LI leg. (SNUC). **Paratypes**: 27 ♂♂, 14 ♀♀, same label data as the holotype (SNUC); 7 ♂♂, **CHINA**: Sichuan Prov. Emeishan City, Mt. Emei, 1.viii.2001, LI & ZHAO leg. (SNUC).

#### Description.

Measurements of males (holotype): BL 3.95–4.17 (4.17); FL 2.89–2.95 (2.95); PL 0.88–0.89 (0.88); EL 1.39–1.50 (1.50); SEL 0.10–0.12 (0.12); HW 0.82–0.84 (0.83); PW 1.38–1.40 (1.39); EW 1.55–1.57 (1.56); relative length of antennomeres I–XI: 23: 14: 15: 10: 13: 14: 15: 12: 14: 13: 26. Measurements of females: BL 4.61–4.89; FL 3.61–3.70; PL 0.89–1.00; EL 1.61–1.72; SEL 0.16–0.18; HW 0.88–0.90; PW 1.49–1.51; EW 1.78–1.83; relative length of antennomeres I–XI: 25: 14: 15: 8: 15: 14: 15: 13: 13: 15: 30.

**Figure 6.**
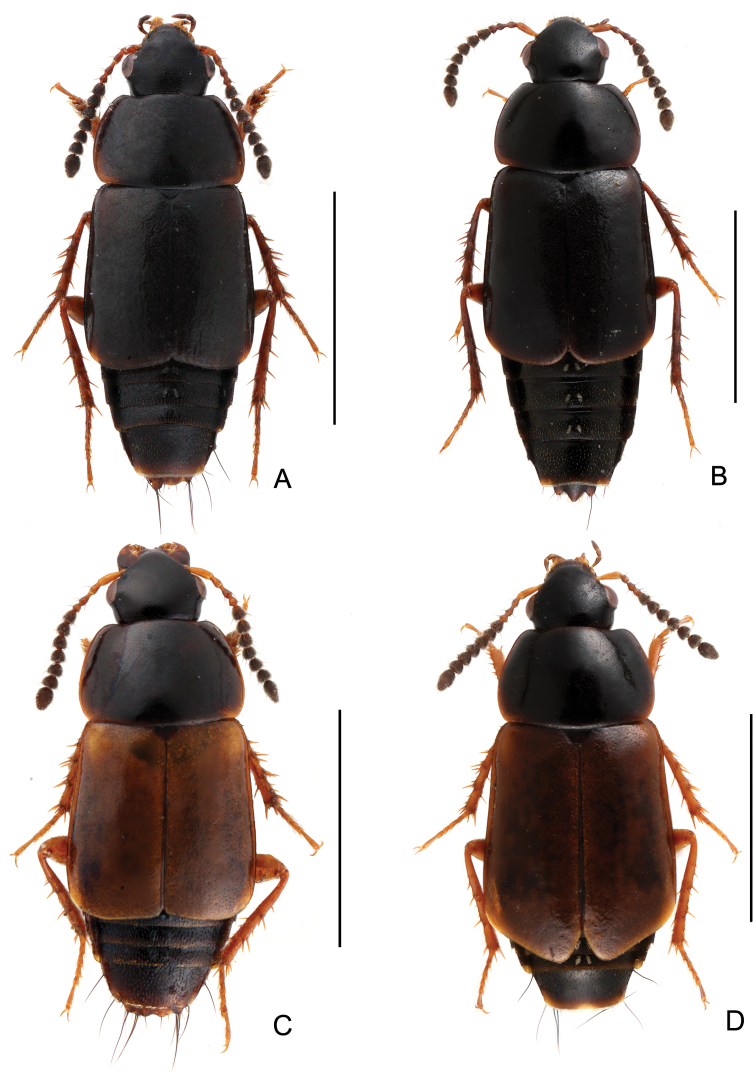
Habitus. **A** *Tachinus oblongoelytratus*, male holotype **B** *Tachinus oblongoelytratus*, female **C** *Tachinus parahercules*, male holotype **D** *Tachinus parahercules*, female. Scales: 2 mm.

Body (Figs 6A, 6B) dark brown to black; head, pronotal disc, elytra and abdomen black; mouthparts, basal four antennomeres, posterior margin of pronotum, posterior margins of posterior abdominal tergites and legs reddish brown.

Head slightly transverse, HW: PW = 0.58–0.61 (0.60); surface with sparse fine punctation, microsculpture consisting of fine striae. Antennomeres X shorter than wide.

Pronotum transverse, PL: PW = 0.59–0.67 (0.63); surface with punctation similar to that of head, microsculpture consisting of transverse striae.

Elytra: EL: PL = 1.56–1.70 (1.70), EL: EW = 1.56–1.70 (0.96), EW: PW = 1.11–1.14 (1.12) in males; EL: PL = 1.61–1.93, EL: EW = 0.88–0.97, EW: PW = 1.18–1.23 in females; apical margin broadly rounded; surface with punctation denser and coarser than that of pronotum, microsculpture consisting of fine transverse meshes.

Male. Sternite VII (Figs 9K, 10K) with semicircular emargination at posterior margin, apical margin bent ventrad, strongly projecting, with large area of coarse granules. Tergite VIII (Fig. 12B) with apical lobes almost fused, forming a sinuate apical margin. Sternite VIII as in Fig. 13K. Median lobe of aedeagus (Fig. 16E) broad and much longer than parameres, apical portion with paired projection.

Female. Elytra only slightly longer than in male, apical margins broadly rounded, only weakly produced near sutural angle. Tergite VIII (Fig. 19C) trilobed, median lobe triangular and broad, lateral lobes short, each with one long seta. Fimbriate median lobes of sternite VIII (Fig. 20L) separated by shallow emargination, sublateral lobes slightly shorter than median ones.

#### Etymology.

The specific name (adjective) is a combination of the Latin adjective “oblongus” and the Greek word “elytron”. It refers to the long elytra.

#### Remarks.

Males of this species can be separated from those of the other species of the group by the unique shape of the aedeagus, females by the long median lobe and the chaetotaxy of the lateral lobes (each with one long seta) of tergite VIII.

### Tachinus (s. str.) parahercules

Feng, Li & Schülke sp. n.

http://zoobank.org/7E4002F7-7564-45AF-91F6-6FFC5CB86818

http://species-id.net/wiki/Tachinus_parahercules

Figs 6C , 6D , 9L , 10L , 12C , 13L , 16F , 19D , 20M , 22C

#### Type locality.

China, Sichuan, Aba A. R., Songpan, Huanglongsi.

#### Type material.

**Holotype**: ♂, **CHINA**: Sichuan Prov., Aba A. R., Songpan county, Huanglongsi, 24.vii.2001, LI & ZHAO leg. (SNUC). **Paratypes**: 20 ♂♂, 18 ♀♀, same label data as the holotype (SNUC); 2 ♀♀, **CHINA**: N–Sichuan [CH12–19] 47 km N Songpan, road S 301 km 118, N Gongangling pass, 33°03'15"N, 103°43'36"E, 3390 m, spruce forest with shrubs, litter, moss, and mushrooms sifted, 9.viii.2012, leg. M. Schülke (cSch); ♀, **CHINA**: [19] N–Sichuan N Songpan, 33°03'15"N, 103°43'36"E, 3390 m, spruce forest, sifted, 9.viii.2012, V. Assing (cAss).

#### Description.

Measurements of males (holotype): BL 3.39–4.23 (4.23); FL 2.89–3.39 (3.39); PL 0.89–1.00 (1.00); EL 1.45–1.50 (1.50); SEL 0.10–0.12 (0.12); HW 0.82–0.84 (0.83); PW 1.33–1.45 (1.45); EW 1.56–1.67 (1.67); relative length of antennomeres I–XI: 24: 15: 15: 8: 15: 13: 13: 13: 13: 13: 26. Measurements of females: BL 4.06–4.45; FL 3.28–3.37; PL 0.95–1.00; EL 1.45–1.62; SEL 0.28–0.33; HW 0.88–0.90; PW 1.45–1.50; EW 1.83–1.89; relative length of antennomeres I–XI: 22: 15: 18: 9: 15: 12: 14: 13: 14: 14: 25.

Body (Figs 6C, 6D) dark brown to black; head black; disc of pronotum reddish brown to dark brown; mouthparts, basal four antennomeres, posterior margin of elytra, and posterior margin of abdominal tergites reddish brown. Sometimes elytra paler and with reddish brown humeral spot.

Head slightly transverse, HW: PW = 0.57–0.63 (0.57); surface with microsculpture consisting of irregular striae, punctation fine and sparse. Antennomeres X distinctly transverse in male, but slightly shorter than wide in female.

Pronotum: PL: PW = 0.61–0.75 (0.69); surface with microsculpture consisting of transverse striae, punctation similar to that of head.

Elytra, EL: PL = 1.45–1.69 (1.50), EL: EW = 0.87–0.96 (0.90), EW: PW = 1.08–1.26 (1.15) in males; EL: PL = 1.45–1.71, EL: EW = 0.77–0.89, EW: PW = 1.22–1.30 in females; punctation denser and coarser than on head and pronotum, microsculpture consisting of transverse meshes.

Abdomen with denser and finer punctation than elytra; surface with microsculpture consisting of transverse striae.

Male. Sternite VII (Figs 9L, 10L) as in *Tachinus cavazzutii*, apical emargination narrower. Tergite VIII as in Fig. 12C, all four lobes nearly fused, forming an almost regularly sinuate apical margin. Sternite VIII as in Fig. 13L. Aedeagus (Fig. 16F) with broad median lobe projecting beyond the apices of the parameres, parameres slightly broader than the median lobe; apical margin of median lobe with projection directed ventrad in lateral view.

Female. Elytra distinctly longer than in male, with inner part of posterior margin distinctly produced, forming a distinct angle. Microsculpture more distinct, consisting of short, irregular, transverse or rhomboid meshes. Apical lobes of tergite VIII (Fig. 19D) almost fused, forming a smooth to sinuate apical margin, lateral angles each with one pair of long setae. Fimbriate median lobes of sternite VIII (Fig. 20M) completely fused, sublateral lobes slightly longer than median one.

#### Etymology.

The specific name is a combination of the prefix “para” and “hercules” (noun in apposition). It alludes to the similarity of this species to *Tachinus hercules*.

#### Remarks.

Males of this new species are similar to those of *Tachinus hercules*, they can be separated from *Tachinus hercules* by the different shape of the apical emargination of sternite VII, and by the size of the parameres. Females are distinguished by the shape of the apical margin of tergite VIII.

### Tachinus (s. str.) paralinzhiensis

Feng & Li sp. n.

http://zoobank.org/DB5CCBA7-4570-4438-9AF6-351D1340B511

http://species-id.net/wiki/Tachinus_paralinzhiensis

Figs 7A , 7B , 9M , 10M , 12D , 14A , 17A , 19E , 20N , 22D

#### Type locality.

China, Tibet A. R., Milin, Duoxiongla Mountain.

#### Type material.

**Holotype**: ♂, **CHINA**: Tibet A. R., Milin County, North Duoxiongla Pass, alt. 3,650–3,800 m, 29.viii.2005, Liang TANG leg. (SNUC). **Paratypes**: 6 ♂♂, 7 ♀♀, same label data as the holotype (SNUC); 26 ♂♂, 12 ♀♀, **CHINA**: Tibet A. R., Linzhi County, Sejila Pass, alt. 4,700 m, 2.viii.2005, Liang TANG leg. (SNUC); 22 ♂♂, 15 ♀♀, **CHINA**: Tibet A. R., Linzhi County, Mt. Sejila, alt. 3,700 m, 5.viii.2005, Liang TANG leg. (SNUC).

#### Description.

Measurements of males (holotype): BL 3.56–3.86 (3.56); FL 2.88–2.90 (2.89); PL 0.83–0.89 (0.89); EL 1.33–1.50 (1.50); SEL 0.11–0.14 (0.11); HW 0.82–0.84 (0.83); PW 1.32–1.34 (1.33); EW 1.44–1.46 (1.45); relative length of antennomeres I–XI: 21: 15: 15: 9: 12: 13: 14: 13: 12: 12: 25. Measurements of females: BL 4.73–5.00; FL 3.39–3.78; PL 0.99–1.01; EL 1.67–1.89; SEL 0.14–0.22; HW 0.89–1.00; PW 1.50–1.67; EW 1.77–1.79; relative length of antennomeres I–XI: 22: 15: 15: 10: 15: 15: 15: 14: 14: 14: 26.

**Figure 7.**
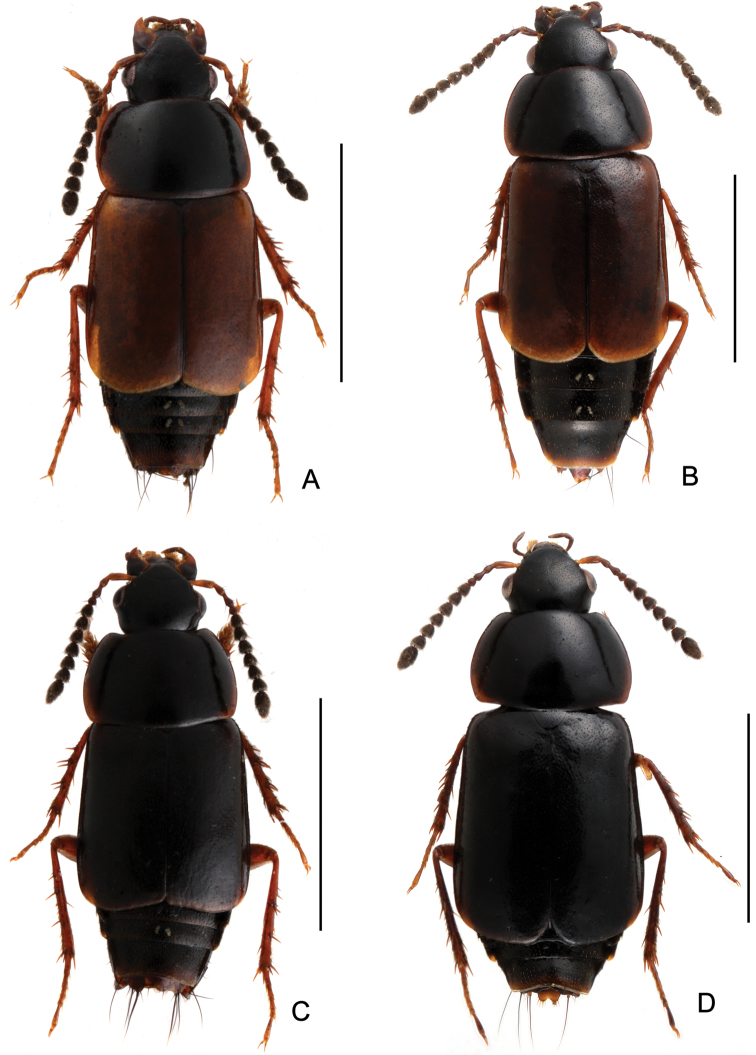
Habitus. **A** *Tachinus paralinzhiensis*, male holotype **B** *Tachinus paralinzhiensis*, female **C** *Tachinus yini*, male holotype **D** *Tachinus yini*, female. Scales: 2 mm.

Body (Figs 7A, 7B) dark brown; mouthparts, segments I–IV of antennae, elytra and legs reddish brown.

Head slightly transverse, HW: PW = 0.53–0.67 (0.62); surface with fine and sparse punctation and microsculpture consisting of irregular striae or meshes. Antennae moderately long, antennomeres X slightly shorter than wide.

Pronotum: PL: PW = 0.59–0.67 (0.67); surface with microsculpture radiating from punctures; punctation finer than that of head.

Elytra: EL: PL = 1.49–1.81 (1.69), EL: EW = 0.91–1.04 (1.03), EW: PW = 1.07–1.11 (1.09) in males; EL: PL = 1.65–1.91, EL: EW = 0.93–1.07, EW: PW = 1.06–1.19 in females; posterior margin broadly rounded. Surface with microsculpture consisting of transverse meshes; punctation coarser than that of pronotum.

Abdomen with coarse punctation, microsculpture consisting of transverse striae.

Male. Posterior margin of sternite VII (Figs 9M, 10M) with broad, shallow median emargination, apical margin bent ventrad, with large area of coarse granules. Tergite VIII (Fig. 12D) with apical lobes almost fused, forming sinuate apical margin. Sternite VIII as in Fig. 14A. Aedeagus (Fig. 17A) similar to that of *Tachinus linzhiensis*, with short median lobe and long parameres. Parameres projecting beyond the apex of the median lobe by one third of the length of the median lobe.

Female. Elytra only slightly longer than in male, posterior margin broadly rounded, with distinct sutural emargination. Abdominal tergite VIII (Fig. 19E) similar to that of *Tachinus linzhiensis*. Sternite VIII (Fig. 20N) with fimbriate median lobes separated by a shallow emargination. Median lobes slightly longer than sublateral ones.

#### Etymology.

The specific name (adjective) is a combination of the Latin prefix “*para*” and “*linzhiensis*”, alluding to the similarity of this species to *Tachinus linzhiensis*.

#### Remarks.

Males are distinguished from those of the other species of the group by the strongly elongate parameres of the aedeagus. Females are distinguished from those of all other species except *Tachinus linzhiensis* by the shape of tergite VIII, from *Tachinus linzhiensis* by the separate fimbriate lobes of sternite VIII.

### Tachinus (s. str.) yini

Feng, Li & Schülke sp. n.

http://zoobank.org/D5B7D6C1-74FD-4E30-9FF1-F29A729D589A

http://species-id.net/wiki/Tachinus_yini

Figs 7C , 7D , 9N , 10N , 12E , 14B , 17B , 19F , 20O , 22E

#### Type locality.

China, Sichuan Province, Luding, Hailuogou Natural Reserve.

#### Type material.

**Holotype**: ♂, **CHINA**: Sichuan Prov. Ganzi A. R., Luding County, Hailuogou, alt. 3,000 m, 21.vii.2006, HU & TANG leg. (SNUC). **Paratypes**: 17 ♂♂, 29 ♀♀, same data as for the holotype (SNUC); 95 ♂♂, 96 ♀♀, **CHINA**: Sichuan Prov., Gonggashan–Hailuogou, 29°35'N, 102°00'E, 2800–3200 m, 3–6.vii.1994, D. Král & J. Farkač leg. (cSme, cSch); 40 ♂♂, 41 ♀♀, **CHINA**: Sichuan Prov., Gongga Shan, Hailuogou, above Camp 3, 3050 m, 29°35'N, 102°00'E, 6.vii.1996, A. Smetana, J. Farkač & P. Kabátek leg. (cSme, cSch); 10 ♂♂, **CHINA**: Sichuan Prov., Gonggashan mts., 29°53'N, 102°01'E, 4000 m, 9.–11.vii.1994, D. Král & J. Farkač leg. (NHMB, cSch); 6 ♂♂, 5 ♀♀, **CHINA**: Sichuan Prov., Gongga Shan, Hailuogou, above Camp 3, 3200 m, 29°35'N, 102°00'E, 7.vii.1996, A. Smetana, J. Farkač & P. Kabátek leg. (cSme, cSch); 2 ♂♂, 1 ♀, **CHINA**: Sichuan Prov., Gongga Shan, Hailuogou, in front of glacier 1, 2850 m, 29°35'N, 102°00'E, 7.vii.1998, A. Smetana leg. (cSme, cSch); 1 ♂, **CHINA**: Sichuan, Gongga Shan, Hailuogou, above Camp 3, 3200 m, 7.VII. 1996 29°35'N, 102°00'E / collected by J. Farkač, P. Kabátek and A. Smetana (NHMB); 23 ♂♂, 12 ♀♀, **CHINA**: W Sichuan, Rte. 138, 15 km W Kangding, 3250 m, 29°57'N, 101°54'E 19.VII.98, J. Král / 1998 China Expedition J. Farkač, D. Král, J. Schneider & A. Smetana (NHMB, cSch).

#### Description.

Measurements of males (holotype): BL 3.61–4.00 (3.61); FL 2.95–3.11 (2.95); PL 0.89–0.95 (0.95); EL 1.50–1.56 (1.56); SEL 0.10–0.12 (0.12); HW 0.83–0.89 (0.89); PW 1.39–1.45 (1.45); EW 1.56–1.61 (1.61); relative length of antennomeres I–XI: 20: 13: 14: 8: 12: 12: 12: 11: 12: 11: 28. Measurements of females: BL 4.06–4.34; FL 3.50–3.67; PL 0.89–1.06; EL 1.50–1.71; SEL 0.27–0.29; HW 0.83–0.89; PW 1.39–1.57; EW 1.56–1.65; relative length of antennomeres I–XI: 20: 14: 15: 9: 13: 13: 14: 12: 13: 14: 27.

Body (Figs 7C, 7D) black; head, antennomeres V–XI, pronotum, disc of elytra and abdomen black; posterior margin of elytra, posterior margin of posterior abdominal tergites and legs reddish brown. Mouthparts and basal four antennomeres sometimes brownish.

Head slightly transverse, HW: PW = 0.53–0.64 (0.61), surface with fine and sparse punctation, sometimes invisible between microsculpture, microsculpture very distinct, consisting of irregular meshes on disc, of more transvers meshes on posterior portion. Ocular setae fine but distinct. Antennomeres X shorter than wide.

Pronotum: PL: PW = 0.57–0.76 (0.66), surface with punctation finer than that of head, microsculpture consisting of transverse meshes.

Elytra, EL: PL = 1.58–1.75 (1.64), EL: EW = 0.93–1.00 (0.97), EW: PW = 1.08–1.16 (1.11) in males; EL: PL = 1.42–1.92, EL: EW = 0.91–1.10, EW: PW = 0.99–1.19 in females; punctation denser and coarser than that of pronotum, microsculpture consisting of short transverse meshes.

Abdomen with punctation coarser than that of head, microsculpture consisting of transverse striae or meshes.

Male. Posterior margin of elytra slightly and evenly emarginate at middle. Sternite VII (Figs 9N, 10N) with apical margin broadly and deeply emarginate; apical margin bent ventrad, with broad area of coarse granules. Tergite VIII (Fig. 12E) distinctly transverse, lobes almost fused, apical margin weakly sinuate. Sternite VIII as in Fig. 14B. Median lobe of aedeagus (Fig. 17B) with pair of ear-shaped apical projections, parameres as broad as the projection of the median lobe, apically truncate.

Female. Elytra distinctly longer than in male, posterior margin broadly rounded, weakly produced near sutural angle. Abdominal tergite VIII (Fig. 19F) with sinuate posterior margin, lateral angles each with only one long seta. Fimbriate median lobes of sternite VIII (Fig. 20O) separated by shallow emargination, median lobes distinctly longer than sublateral ones.

#### Etymology.

The species is named after Zi-Wei Yin (Shanghai), for his various help during this study.

#### Remarks.

Males of this species can bes eparated from those of the other species by the aedeagal median lobe with broad ear-shaped apical projections. Females are distinguished by the sinuate posterior margin and the chaetotaxy of the lateral angles (each with only one long seta) of tergite VIII, from those of *Tachinus maderianus* by the shape of the sutural angle of the elytra.

### Key to males (excluding *Tachinus maderi*)

**Table:** 

1	Lateral margins of pronotum broadly yellow (Figs 3C, 4C, 4D). Sternite VII with comb-like setae at sides of posterior margin (Figs 9F, 9H). Sternite VIII with less distinctly produced median portion of anterior margin, emargination at posterior margin deep, bell-shaped (Figs 13F, 13H)	2
–	Lateral margins of pronotum not broadly yellow (Figs 1–2, 3A, 3B, 4A, 4B, 5–7). Sternite VII without comb-like setae. Sternite VIII with distinctly produced median portion of anterior margin, emargination at posterior margin less deep and with small median incision (Figs 13A–E, 13G, 14A–B)	3
2	Parameres in lateral view slightly curved ventrad in apical half, apices more evenly rounded in ventral view (Fig. 16B). Western Sichuan	*Tachinus lohsei*
–	Parameres in lateral view straight, not curved ventrad in apical half, apices angled at apical ¼ in ventral view (Fig. 15F). Northern Sichuan	*Tachinus jiuzhaigouensis*
3	Parameres distinctly projecting beyond median lobe of aedeagus (Figs 16A, 17A)	4
–	Parameres not projecting beyond median lobe of aedeagus (Figs 15, 16B, 16C, 16D, 16E, 16F, 17B)	5
4	Aedeagus as in Fig. 16A. Xizang	*Tachinus linzhiensis*
–	Aedeagus as in Fig. 17A. Xizang	*Tachinus paralinzhiensis*
5	Parameres small and short (Fig. 16E), their apices not directed laterad. Sichuan (Emei Shan).	*Tachinus oblongoelytratus*
–	Parameres larger, their apices of more lateral (Figs 15A, 15B, 15C, 16C, 16D, 16F, 17B) or latero-apical (Figs 15D, 15E) orientation	6
6	Parameres forming a V, oblique, combined width greater than that of median lobe, reaching apex of median lobe (Fig. 15D). Northern Sichuan	*Tachinus hercules*
–	Parameres not forming a V, if combined width greater than that of median lobe, not reaching apex of median lobe	7
7	Parameres broad, 1.7 times as wide as basal part of median lobe (Fig. 17B), apical part of median lobe distinctly widened. Western Sichuan	*Tachinus yini*
–	Parameres 1.3 times as wide as basal part of median lobe at most, apical part of median lobe not distinctly widened	8
8	Posterior margin of sternite VII with deep and broad semicircular emargination (Fig. 9I). Apical margin of parameres truncate (Fig. 16C). Southern Sichuan	*Tachinus maderianus*
–	Posterior margin of sternite VII less deeply and broadly emarginate	9
9	Parameres with basal portion of reduced length, shorter than 1/3 of median lobe (Fig. 15A), forming one apically truncate plate. Northern Sichuan	*Tachinus armatus*
–	Basal part of parameres not of reduced length, parameres longer than 1/3 of median lobe	10
10	Posterior margin of sternite VII with broad triangular emargination (Figs 9C, 9E, 9J)	11
–	Posterior margin of sternite VII without triangular emargination (Figs 9B, 9L)	13
11	Median lobe of aedeagus in lateral view with acute projection near apex of ventral side (Figs 15C, 15E)	12
–	Median lobe of aedeagus in lateral view without acute projection (Fig. 16D). Qinghai, Gansu, Northern Sichuan	*Tachinus mengdaensis*
12	Projection near apex of ventral side of median lobe (lateral view) small (Fig. 15E), apex of median lobe with larger emargination in ventral view. Shaanxi (Qinling Shan)	*Tachinus hujiayaoi*
–	Projection near apex of ventral side of median lobe (lateral view) robust (Fig. 15C), apex of median lobe with smaller emargination in ventral view. Ningxia, Qinghai	*Tachinus coronatus*
13	Bent portion of sternite VII distinctly broader than 1/3 of the posterior margin (posterior view) (Fig. 10B). Aedeagus as in Fig. 15B. Northern Sichuan	*Tachinus cavazzutii*
–	Bent portion of sternite VII not distinctly broader than 1/3 of the posterior margin (posterior view) (Fig. 10L). Aedeagus as in Fig. 16F. Northern Sichuan	*Tachinus parahercules*

### Key to females

**Table:** 

1	Lateral margins of pronotum broadly yellowish brown, distinctly contrasting with the dark disc (Figs 3C, 3D, 4C, 4D). Median lobe of tergite VIII separated from lateral lobes by deep incision or suture (Fig. 18H, 18F). Median lobes of sternite VIII long and slender, distinctly separated by a deep emargination (Figs 20H, 20F)	2
–	Lateral margins of pronotum not or only narrowly paler than disc. Median and lateral lobes of tergite VIII more or less fused, at most separated apically (Figs 18G or 19E). Median lobes of sternite VIII fused or separated only by a shallow emargination	3
2	Median lobe of tergite VIII shorter than lateral lobes (Fig. 18H). Western Sichuan	*Tachinus lohsei*
–	Median lobe of tergite VIII as long as or longer than lateral lobes (Fig. 18F). Northern Sichuan	*Tachinus jiuzhaigouensis*
3	Tergite VIII not strongly transverse, at base less than 1.5 times as wide as long (Figs 18G, 19E, 19C)	4
–	Tergite VIII strongly transverse, at base more than 1.5 times as wide as long (Figs 18A–E, 18I, 19A–B, 19D–F)	6
4	Lateral lobes of tergite VIII each with one long setae, median lobe of obtusely triangular shape (Fig. 19C). Sichuan (Emei Shan)	*Tachinus oblongoelytratus*
–	Lateral lobes of tergite VIII each with a pair of long setae, median lobe with shallow apical emargination (Figs 18G, 19E)	5
5	Fimbriate median lobes of sternite VIII fused (Fig. 20G). Xizang	*Tachinus linzhiensis*
–	Fimbriate median lobes of sternite VIII not fused, separated by small and shallow emargination (Fig. 20N). Xizang	*Tachinus paralinzhiensis*
6	Lateral lobes or angles of tergite VIII each with only one long seta (Figs 18B, 18D, 19A, 19B, 19F)	7
–	Lateral lobes or angles of tergite VIII each with two long setae (Figs 18A, 18E, 19D, 18C, 18I). Species reliably distinguished only based on the male sexual characters	11
7	Apical margin of tergite VIII with broad and deep emargination (Fig. 18B). Northern Sichuan	*Tachinus cavazzutii*
–	Apical margin of tergite VIII more or less straight (Figs 19A, 19B, 19F) or distinctly produced (Figs 18D)	8
8	Apical margin of tergite VIII distinctly produced at middle (Fig. 18D). Northern Sichuan	*Tachinus hercules*
–	Apical margin of tergite VIII not distinctly produced at middle	9
9	Apical margin of tergite VIII more or less straight at middle (Fig. 19A). Inner part of posterior margin of elytra produced, forming a distinct angle (Fig. 5B). Southern Sichuan	*Tachinus maderianus*
–	Apical margin of tergite VIII sinuate or weakly produced at middle (Figs 19B, 19F). Inner part of posterior margin of elytra not produced, posterior margin more or less evenly rounded (Fig. 5D, 7D)	10
10	Apical margin of tergite VIII sinuate (Fig. 19F). Western Sichuan	*Tachinus yini*
–	Apical margin of tergite VIII weakly produced at middle (Fig. 19B). Qinghai, Gansu, Northern Sichuan	*Tachinus mengdaensis*
11	Apical margin of tergite VIII produced at middle (Figs 18E, 18C). Fimbriate median lobes of sternite VIII nearly fused (Figs 20E, 20M). Inner part of posterior margin of elytra not produced (Figs 2B, 3B)	12
–	Apical margin of tergite VIII straight, sinuate or weakly emarginate at middle (Figs 18A, 18I, 19D). Inner part of posterior margin of elytra produced, forming distinct angle (Figs 1B, 6D, 8A)	13
12	Tergite VIII as in Fig. 18E. Sternite VIII as in Fig. 20E. Shaanxi (Qinling Shan)	*Tachinus hujiayaoi*
–	Tergite VIII as in Fig. 18C. Sternite VIII as in Fig. 20C. Ningxia, Qinghai	*Tachinus coronatus*
13	Posterior margin of tergite VIII sinuate and distinctly emarginate at middle, postero-lateral angles of tergite VIII broadly rounded (Fig. 19D). Fimbriate median lobes of sternite VIII fused (Fig. 20M). Northern Sichuan	*Tachinus parahercules*
–	Posterior margin of tergite VIII straight or sinuate at middle, postero-lateral angles of tergite VIII more acute (Figs 18A, 18I)	14
14	Posterior margin of tergite VIII nearly straight (Fig. 18A). Northern Sichuan	*Tachinus armatus*
–	Posterior margin of tergite VIII distinctly sinuate (Fig. 18I). Western Sichuan	*Tachinus maderi*

**Figure 8.**
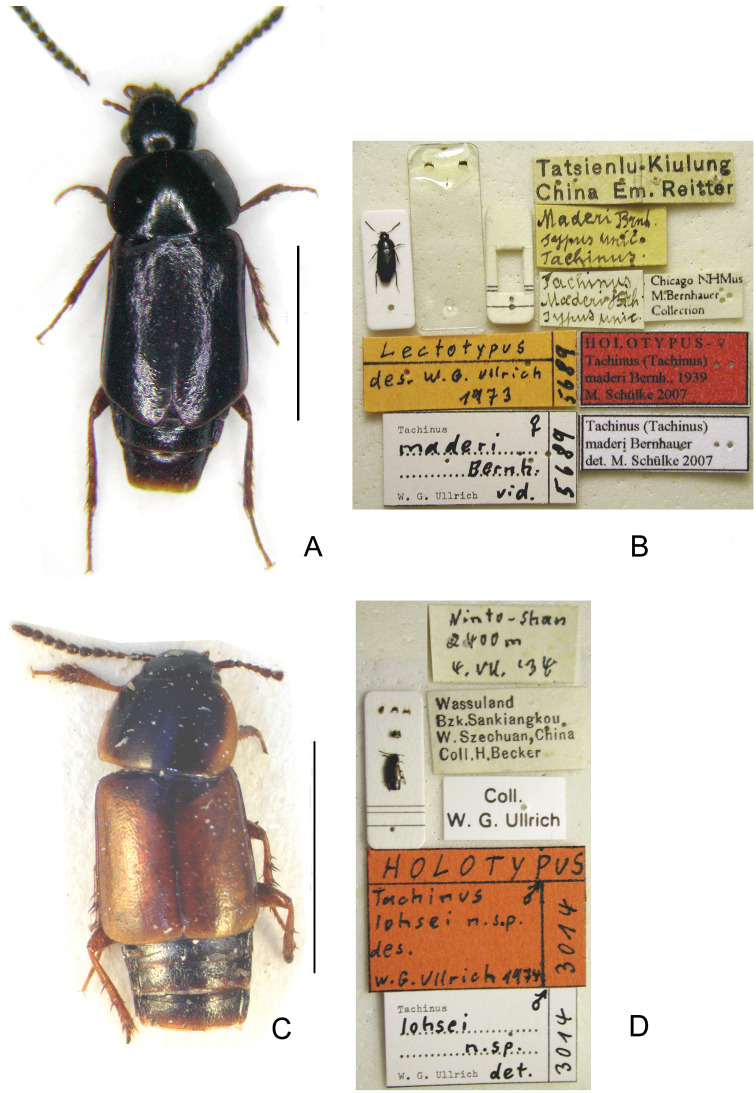
Habitus and labels. **A** *Tachinus maderi*, female holotype, habitus **B** *Tachinus maderi*, female holotype, labels **C** *Tachinus lohsei*, male holotype, habitus **D** *Tachinus lohsei*, male holotype, labels. Scales: 2 mm.

**Figure 9.**
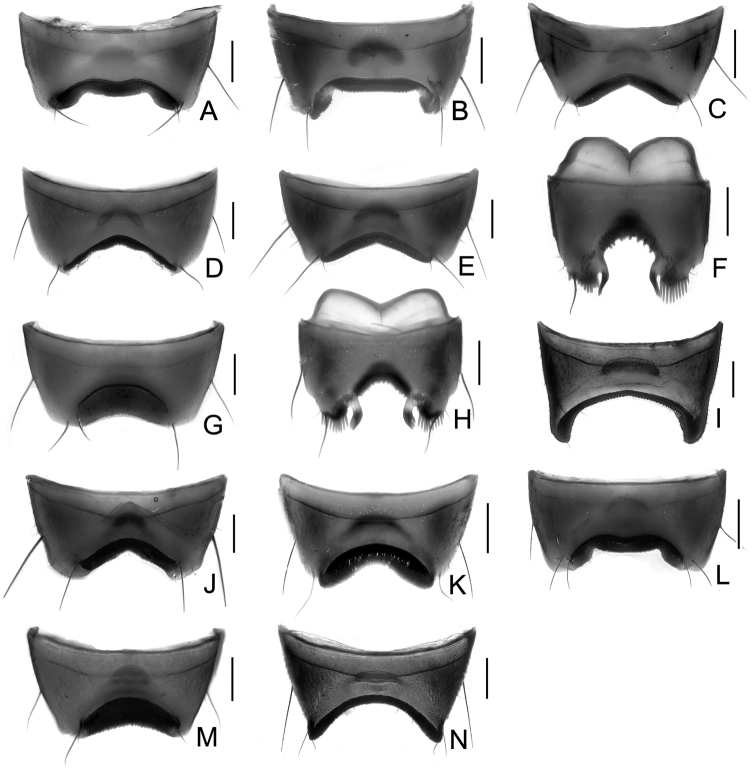
Ventral view of male sternite VII. **A** *Tachinus armatus* **B** *Tachinus cavazzutii* **C** *Tachinus coronatus* **D** *Tachinus hercules* **E** *Tachinus hujiayaoi* **F** *Tachinus jiuzhaigouensis* **G** *Tachinus linzhiensis* **H** *Tachinus lohsei* **I** *Tachinus maderianus* **J** *Tachinus mengdaensis* **K** *Tachinus oblongoelytratus* **L** *Tachinus parahercules* **M** *Tachinus paralinzhiensis* **N** *Tachinus yini*. Scales: 0.2 mm.

**Figure 10.**
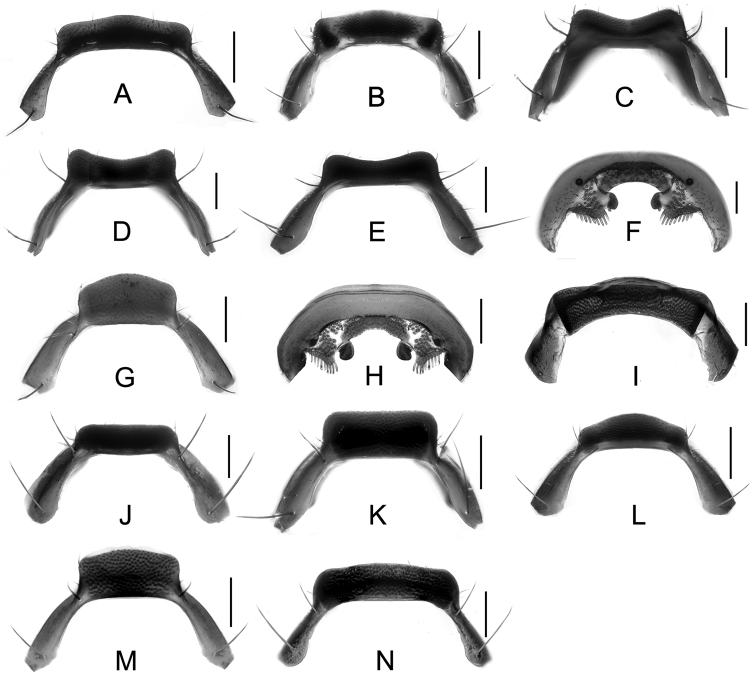
Posterior view of male sternite VII. **A** *Tachinus armatus* **B** *Tachinus cavazzutii* **C** *Tachinus coronatus* **D** *Tachinus hercules* **E** *Tachinus hujiayaoi* **F** *Tachinus jiuzhaigouensis* **G** *Tachinus linzhiensis* **H** *Tachinus lohsei* **I** *Tachinus maderianus* **J** *Tachinus mengdaensis* **K** *Tachinus oblongoelytratus* **L** *Tachinus parahercules* **M** *Tachinus paralinzhiensis* **N** *Tachinus yini*. Scales: 0.2 mm.

**Figure 11.**
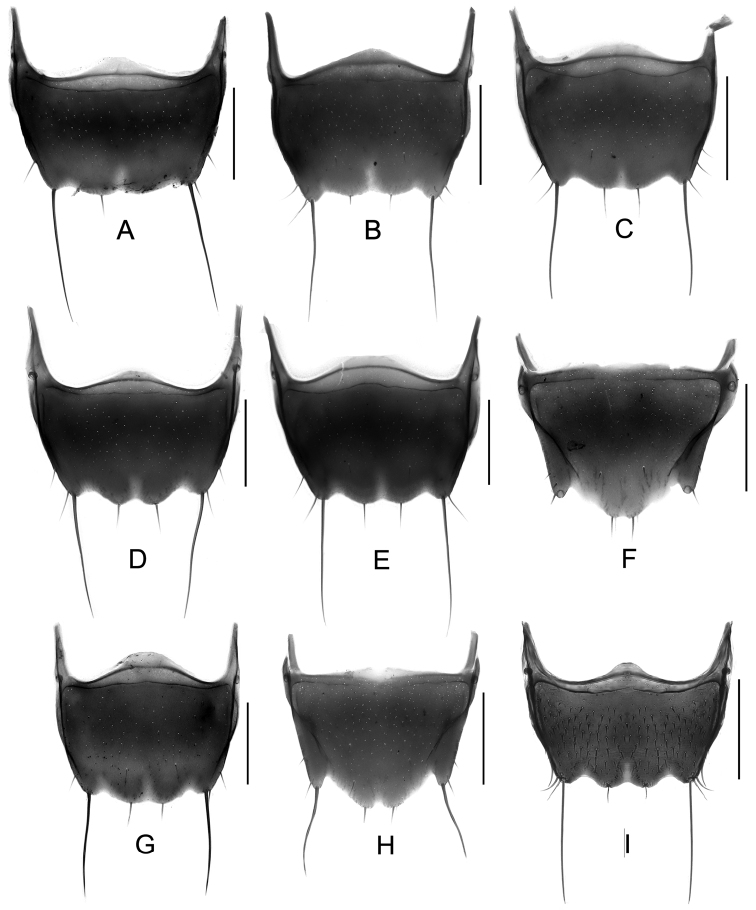
Dorsal view of male tergite VIII. **A** *Tachinus armatus* **B** *Tachinus cavazzutii* **C** *Tachinus coronatus* **D** *Tachinus hercules* **E** *Tachinus hujiayaoi* **F** *Tachinus jiuzhaigouensis* **G** *Tachinus linzhiensis* **H** *Tachinus lohsei* **I** *Tachinus maderianus*. Scales: 0.3 mm.

**Figure 12.**
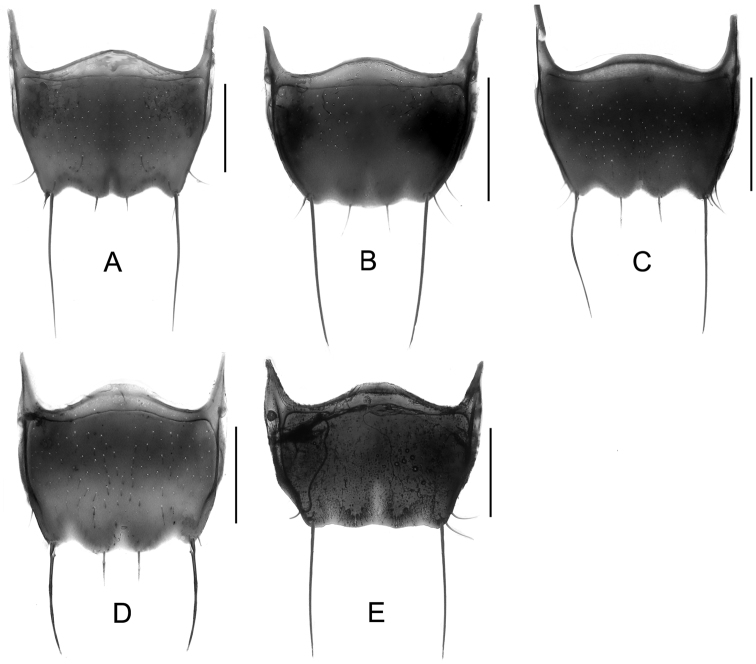
Dorsal view of male tergite VIII. **A** *Tachinus mengdaensis* **B** *Tachinus oblongoelytratus* **C** *Tachinus parahercules* **D** *Tachinus paralinzhiensis* **E** *Tachinus yini*. Scales: 0.3 mm.

**Figure 13.**
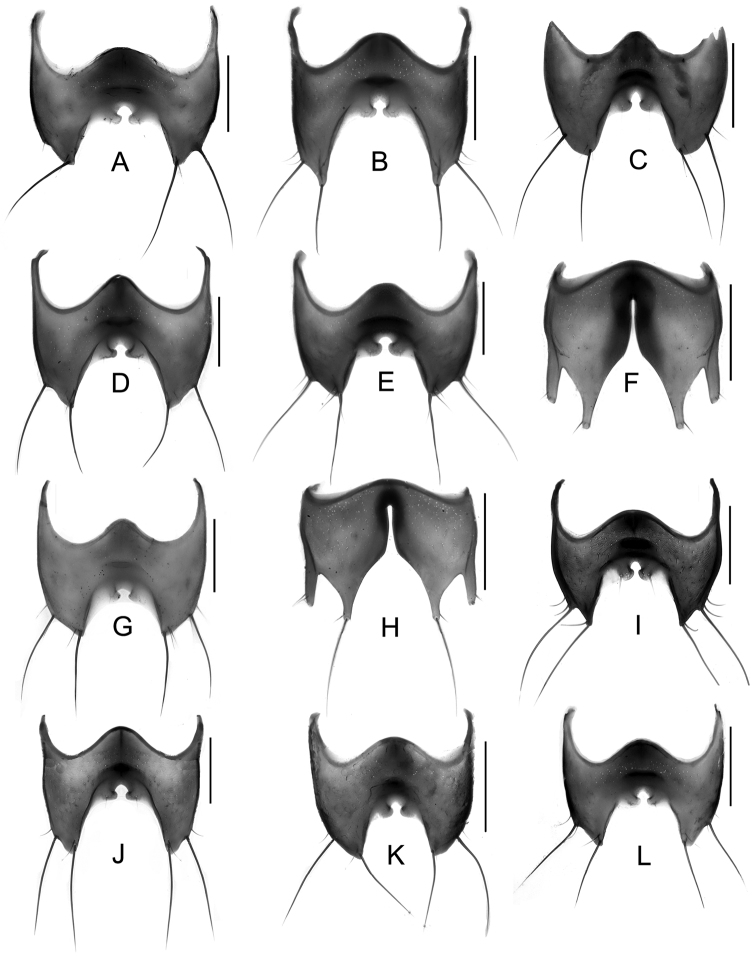
Ventral view of male sternite VIII. **A** *Tachinus armatus* **B** *Tachinus cavazzutii* **C** *Tachinus coronatus* **D** *Tachinus hercules* **E** *Tachinus hujiayaoi* **F** *Tachinus jiuzhaigouensis* **G** *Tachinus linzhiensis* **H** *Tachinus lohsei* **I** *Tachinus maderianus* **J** *Tachinus mengdaensis* **K** *Tachinus oblongoelytratus* **L** *Tachinus parahercules*. Scales: 0.3 mm.

**Figure 14.**
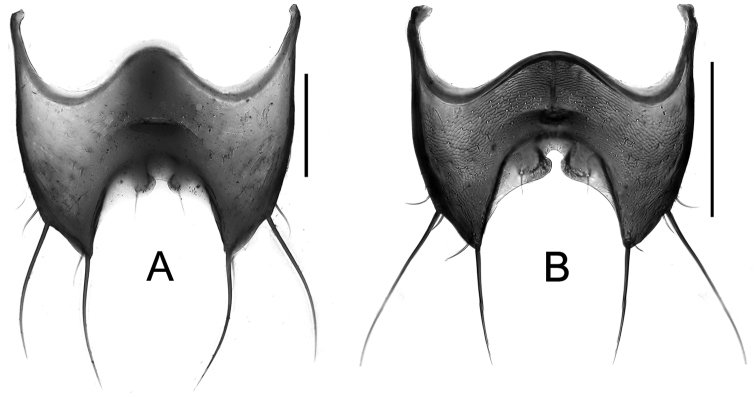
Ventral view of male sternite VIII. **A** *Tachinus paralinzhiensis* **B** *Tachinus yini*. Scales: 0.3 mm.

**Figure 15.**
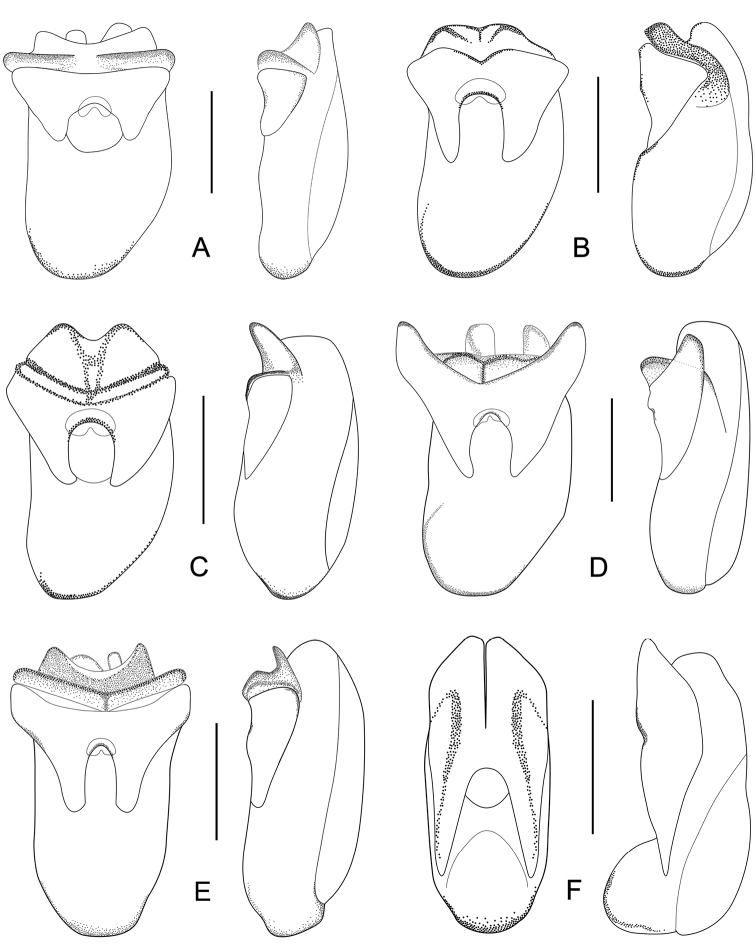
Ventral and lateral view of aedeagus (left: ventral view; right: lateral view). **A** *Tachinus armatus* **B** *Tachinus cavazzutii* **C** *Tachinus coronatus* **D** *Tachinus hercules* **E** *Tachinus hujiayaoi* **F** *Tachinus jiuzhaigouensis*. Scales: 0.3 mm.

**Figure 16.**
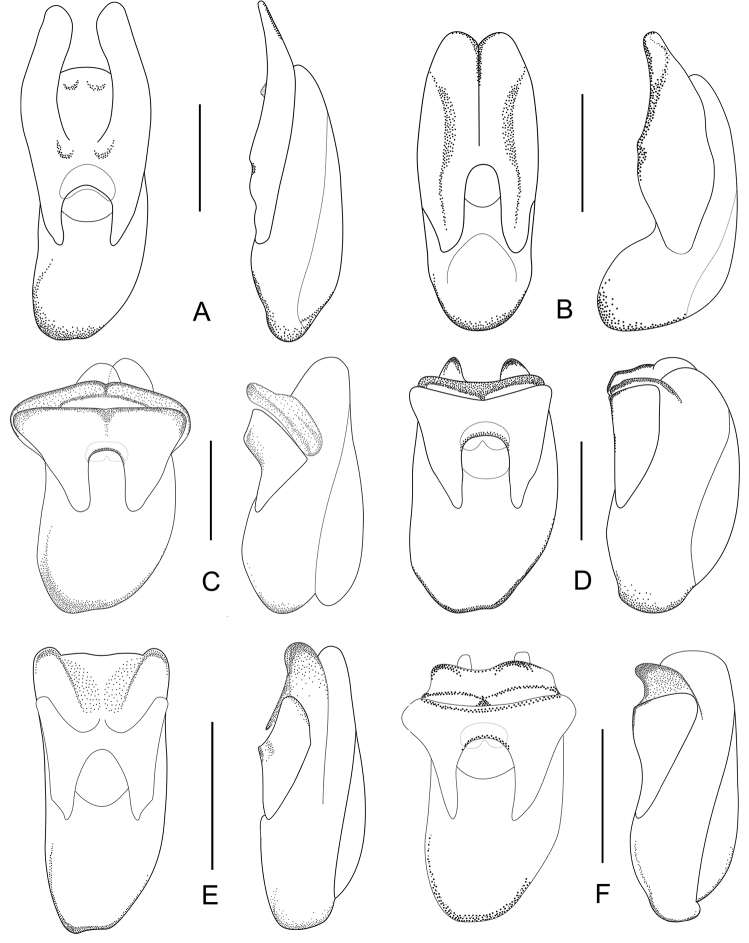
Ventral and lateral view of aedeagus (left: ventral view; right: lateral view). **A** *Tachinus linzhiensis* **B** *Tachinus lohsei* **C** *Tachinus maderianus* **D** *Tachinus mengdaensis* **E** *Tachinus oblongoelytratus* **F** *Tachinus parahercules*. Scales: 0.3 mm.

**Figure 17.**
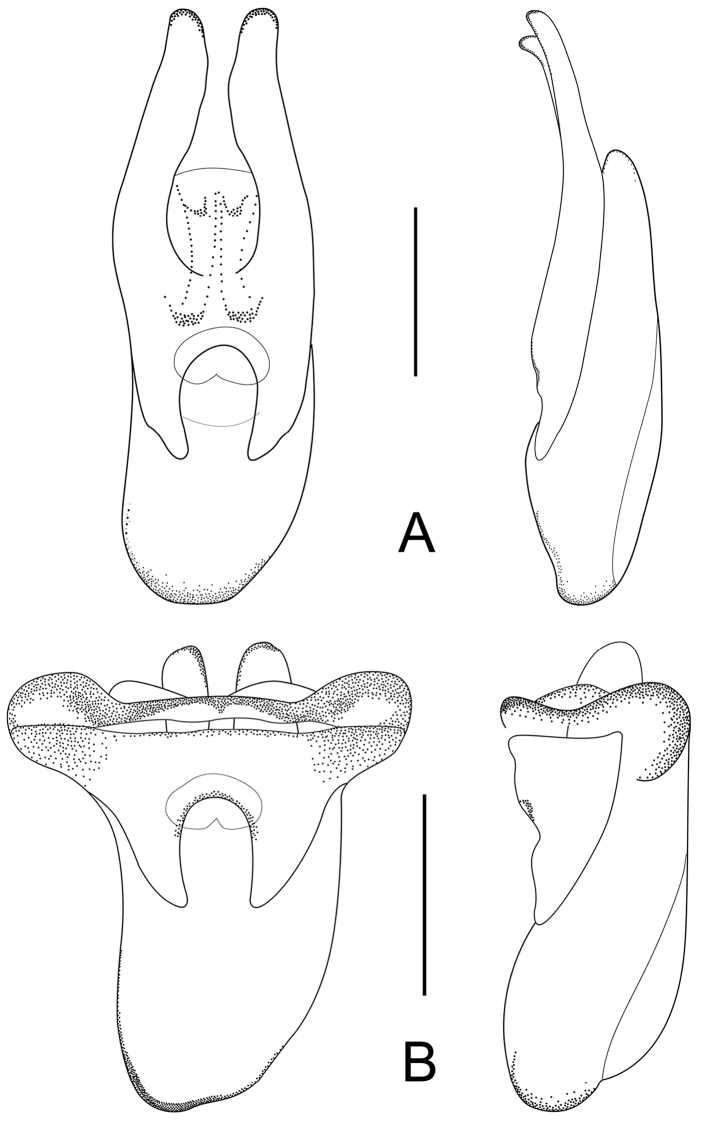
Ventral and lateral view of aedeagus (left: ventral view; right: lateral view). **A** *Tachinus paralinzhiensis* **B** *Tachinus yini*. Scales: 0.3 mm.

**Figure 18.**
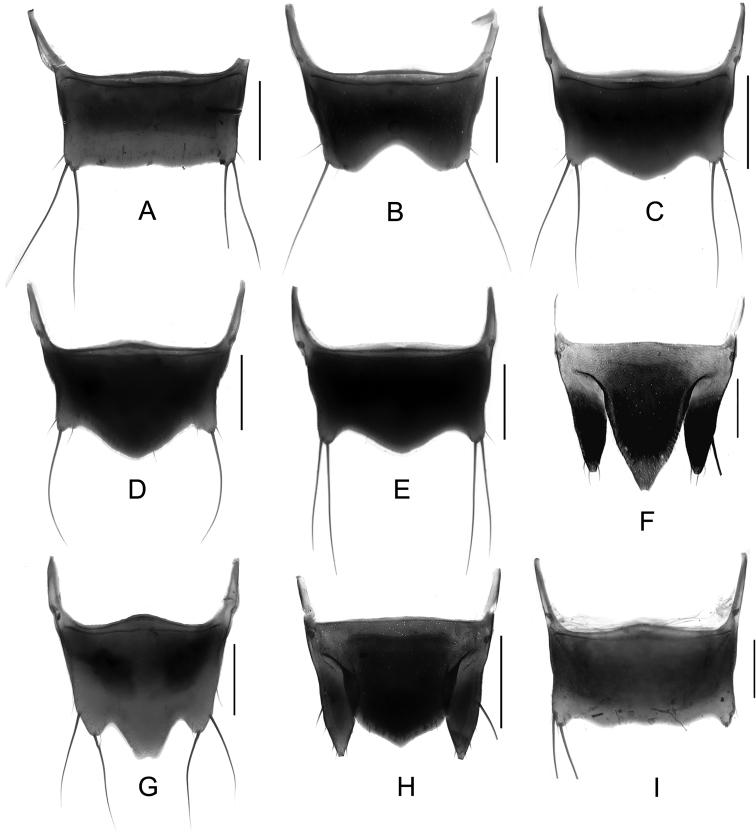
Ventral view of female tergite VIII. **A** *Tachinus armatus* **B** *Tachinus cavazzutii* **C** *Tachinus coronatus* **D** *Tachinus hercules* **E** *Tachinus hujiayaoi* **F** *Tachinus jiuzhaigouensis* **G** *Tachinus linzhiensis* **H** *Tachinus lohsei* **I** *Tachinus maderi*. Scales: 0.2 mm.

**Figure 19.**
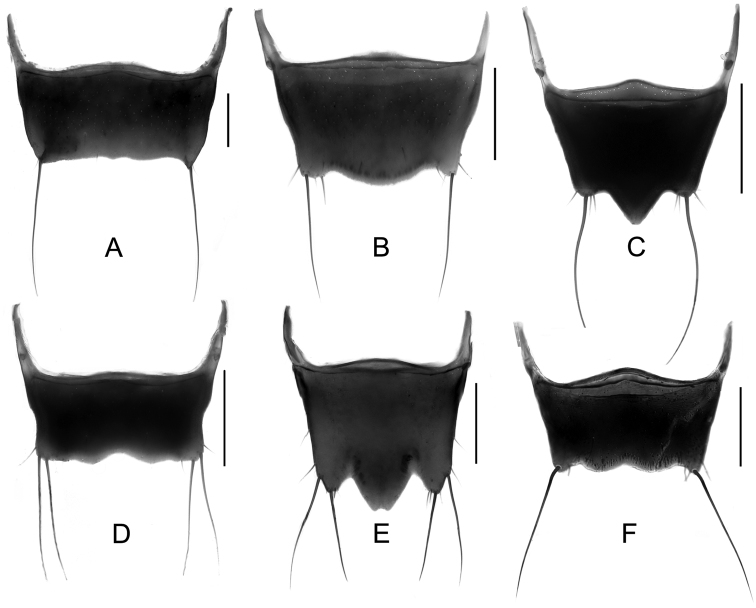
Ventral view of female tergite VIII. **A** *Tachinus maderianus* **B** *Tachinus mengdaensis* **C** *Tachinus oblongoelytratus* **D** *Tachinus parahercules* **E** *Tachinus paralinzhiensis* **F** *Tachinus yini*. Scales: 0.2 mm.

**Figure 20.**
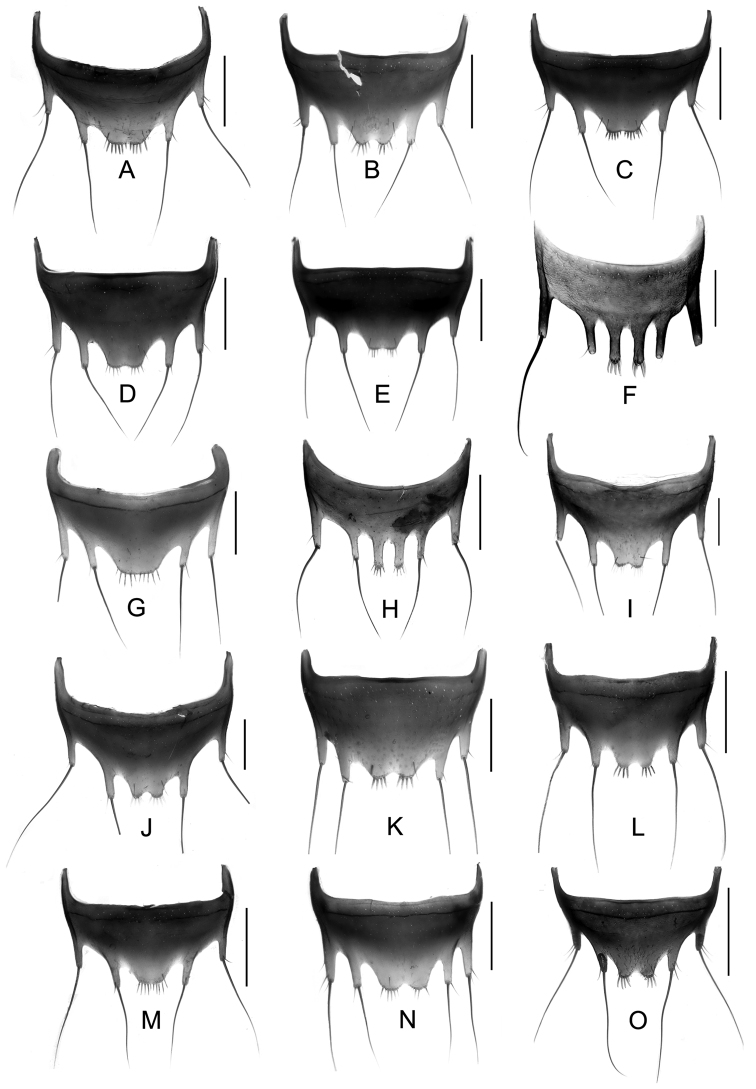
Ventral view of female sternite VIII. **A** *Tachinus armatus* **B** *Tachinus cavazzutii* **C** *Tachinus coronatus* **D** *Tachinus hercules* **E** *Tachinus hujiayaoi* **F** *Tachinus jiuzhaigouensis* **G** *Tachinus linzhiensis* **H** *Tachinus lohsei* **I** *Tachinus maderi* **J** *Tachinus maderianus* **K** *Tachinus mengdaensis* **L** *Tachinus oblongoelytratus* **M** *Tachinus parahercules* **N** *Tachinus paralinzhiensis* **O** *Tachinus yini*. Scales: 0.2 mm.

**Figure 21.**
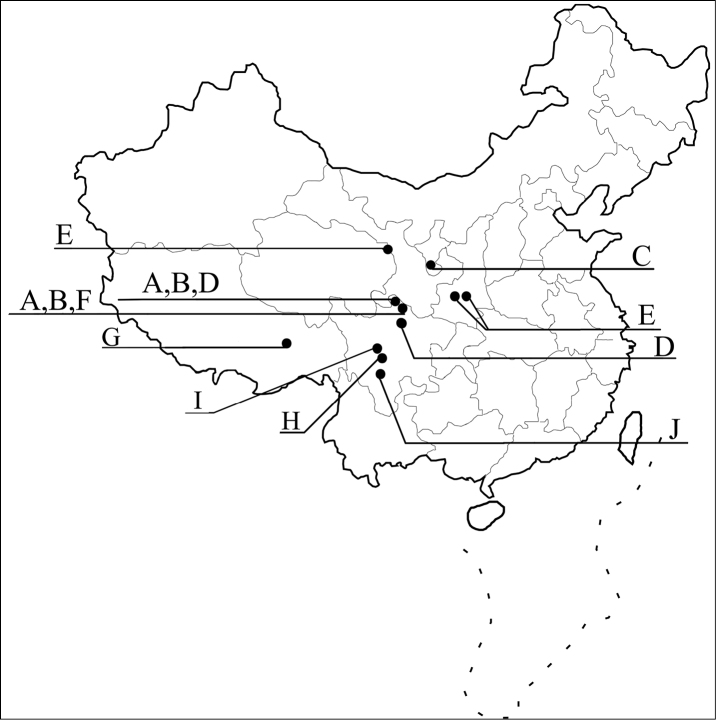
Distribution of the *silphaeformis* species group in China. **A** *Tachinus armatus* **B** *Tachinus cavazzutii* **C** *Tachinus coronatus* **D** *Tachinus hercules* **E** *Tachinus hujiayaoi* **F** *Tachinus jiuzhaigouensis* **G** *Tachinus linzhiensis* **H** *Tachinus lohsei* **I** *Tachinus maderi* **J** *Tachinus maderianus*.

**Figure 22.**
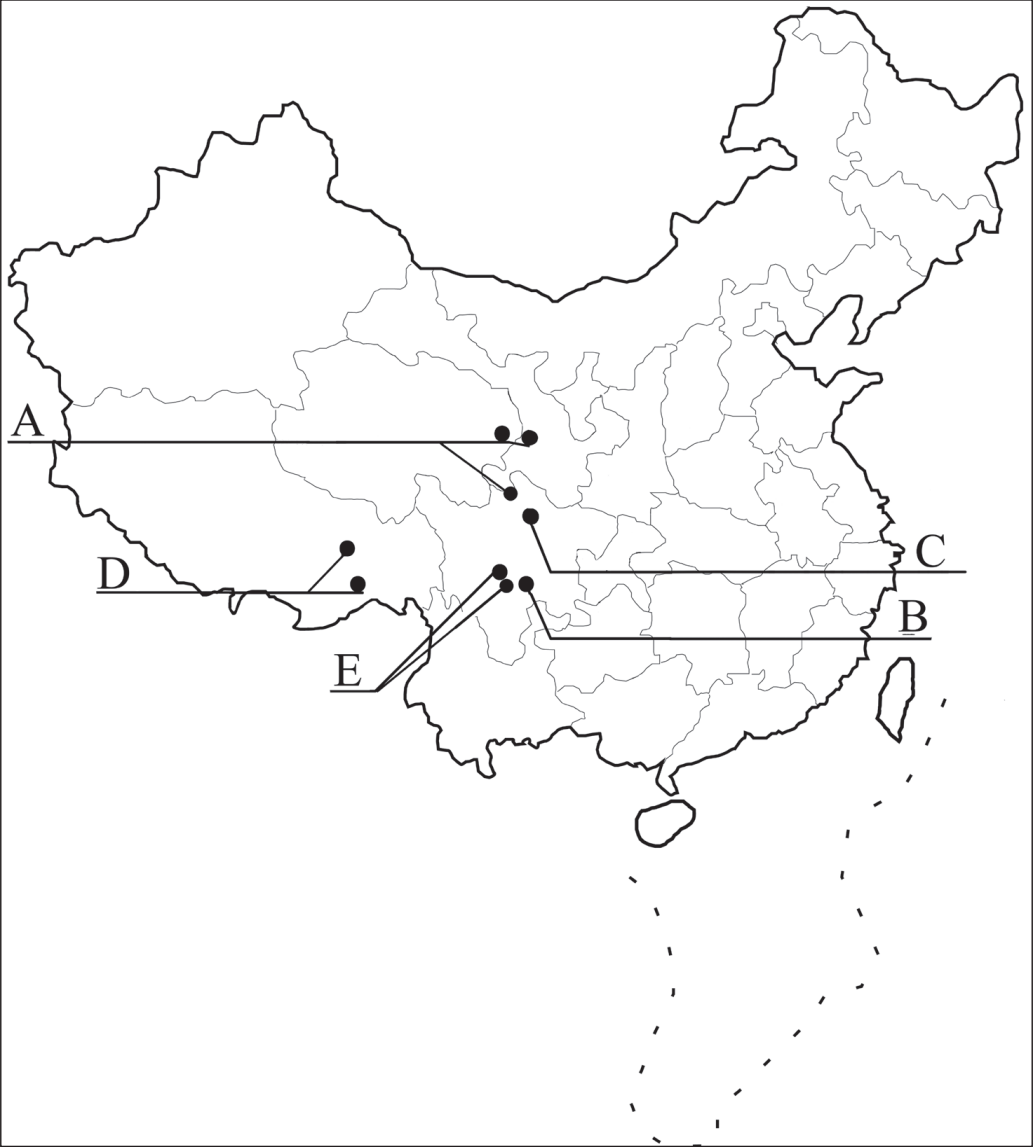
Distribution of the *silphaeformis* species group in China. **A** *Tachinus mengdaensis* **B** *Tachinus oblongoelytratus* **C** *Tachinus parahercules* **D** *Tachinus paralinzhiensis* **E** *Tachinus yini*.

## Supplementary Material

XML Treatment for Tachinus (s. str.) armatus

XML Treatment for Tachinus (s. str.) cavazzutii

XML Treatment for Tachinus (s. str.) coronatus

XML Treatment for Tachinus (s. str.) hercules

XML Treatment for Tachinus (s. str.) hujiayaoi

XML Treatment for Tachinus (s. str.) jiuzhaigouensis

XML Treatment for Tachinus (s. str.) linzhiensis

XML Treatment for Tachinus (s. str.) lohsei

XML Treatment for Tachinus (s. str.) maderi

XML Treatment for Tachinus (s. str.) maderianus

XML Treatment for Tachinus (s. str.) mengdaensis

XML Treatment for Tachinus (s. str.) oblongoelytratus

XML Treatment for Tachinus (s. str.) parahercules

XML Treatment for Tachinus (s. str.) paralinzhiensis

XML Treatment for Tachinus (s. str.) yini

## References

[B1] Bernhauer M (1939) Zur Staphylinidenfauna von China u. Japan (11. Beitrag). Entomologisches Nachrichtenblatt (Troppau) 12(3/4) (1938): 145–158

[B2] Eppelsheim E (1889) Neue Staphylinen aus den Kaukasusländern, besonders aus Circassien. Wiener Entomologische Zeitung 8: 11-22

[B3] Gravenhorst JLC (1802) Coleoptera Microptera Brunsvicensia nec non exoticorum quotquot exstant in collectionibus entomologorum Brunsvicensium in genera familias et species distribuit. Carolus Reichard, Brunsuigae, lxvi + 206 pp.

[B4] Herman LH (2001) Catalog of the Staphylinidae (Insecta, Coleoptera). 1758 to the end of the second millennium. II., Tachyporine Group. Bulletin of the American Museum of Natural History 265: 651-1066

[B5] Jarrige J (1966) Un *Tachinus* nouveau d'Italie. L'Entomologiste 21(6) (1965): 99–101

[B6] Motschulsky V (1858) Enumération des nouvelles espèces de Coléoptères rapportés de ses voyages (Continuation). Bulletin de la Société Impériale des Naturalistes de Moscou 31: 204–264

[B7] Normand H (1928) Nouveaux coléoptères de la faune tunisienne (17^e^ note). Bulletin de la Société Entomologique de France 1928: 115-118

[B8] Scheerpeltz O (1976) Wissenschaftliche Ergebnisse entomologischer Aufsammlungen in Nepal (Col. Staphylinidae). Khumbu Himal 5: 77-173

[B9] Schülke M (1997) Beitrag zur Systematik der Gattung *Tachinus* Gravenhorst, 1802 (Insecta: Coleoptera: Staphylinidae: Tachyporinae). Reichenbachia 32: 41-48

[B10] Ullrich WG (1975) Monographie der Gattung *Tachinus* Gravenhorst (Coleoptera: Staphylinidae), mit Bemerkungen zur Phylogenie und Verbreitung der Arten. Christian-Albrechts-Universität, Kiel, 365 pp., LXI pl

